# Photooxidation and Pentagalloyl Glucose Cross-Linking Improves the Performance of Decellularized Small-Diameter Vascular Xenograft *In Vivo*

**DOI:** 10.3389/fbioe.2022.816513

**Published:** 2022-03-24

**Authors:** Yuhong Liu, Chunyang Chen, Xinlong Xie, Haoyong Yuan, Zhenjie Tang, Tao Qian, Yalin Liu, Mingzhe Song, Sixi Liu, Ting Lu, Zhongshi Wu

**Affiliations:** ^1^ Department of Cardiovascular Surgery, The Second Xiangya Hospital of Central South University, Changsha, China; ^2^ NHC Key Laboratory of Birth Defect for Research and Prevention, Hunan Provincial Maternal and Child Health Care Hospital, Changsha, China

**Keywords:** small-diameter vascular graft, bovine internal mammary artery, decellularization, cross-linking, neointima, tissue engineering vascular graft

## Abstract

Small-diameter vascular grafts have a significant need in peripheral vascular surgery and procedures of coronary artery bypass graft (CABG); however, autografts are not always available, synthetic grafts perform poorly, and allografts and xenografts dilate, calcify, and induce inflammation after implantation. We hypothesized that cross-linking of decellularized xenogeneic vascular grafts would improve the mechanical properties and biocompatibility and reduce inflammation, degradation, and calcification *in vivo*. To test this hypothesis, the bovine internal mammary artery (BIMA) was decellularized by detergents and ribozymes with sonication and perfusion. Photooxidation and pentagalloyl glucose (PGG) were used to cross-link the collagen and elastin fibers of decellularized xenografts. Modified grafts’ characteristics and biocompatibility were studied *in vitro* and *in vivo*; the grafts were implanted as transposition grafts in the subcutaneous of rats and the abdominal aorta of rabbits. The decellularized grafts were cross-linked by photooxidation and PGG, which improved the grafts’ biomechanical properties and biocompatibility, prevented elastic fibers from early degradation, and reduced inflammation and calcification *in vivo*. Short-term aortic implants in the rabbits showed collagen regeneration and differentiation of host smooth muscle cells. No occlusion and stenosis occurred due to remodeling and stabilization of the neointima. A good patency rate (100%) was maintained. Notably, implantation of non-treated grafts exhibited marked thrombosis, an inflammatory response, calcification, and elastin degeneration. Thus, photooxidation and PGG cross-linking are potential tools for improving grafts’ biological performance within decellularized small-diameter vascular xenografts.

## Introduction

Cardiovascular disease (CVD) is a serious disease that threatens human health. According to the World Health Organization, more than 17 million people die of CVD every year (Organization, 2013). Nearly 400,000 procedures of coronary artery bypass grafts (CABGs) are performed in the Unites States, and 50,000 procedures are performed in China each year, and there are still many patients waiting for surgery (Writing Group et al., 2016; Ma et al., 2020). The use of the grate saphenous vein, internal mammary artery, and radial artery is the most suitable choice for CABG procedures and peripheral vascular surgery, but some patients have insufficient access to suitable autologous blood vessels due to trauma, varices, infection reoperation, etc. (Wang et al., 2007; Desai et al., 2011).

With the development of tissue engineering vascular grafts, a variety of biodegradable and non-biodegradable synthetic polymers are widely used in reconstructive operations. Polymers, such as expanded polytetrafluoroethylene (ePTFE, Gore-Tex) and polyethylene terephthalate (PET, Dacron), are widely used for large diameter applications (> 6 mm) (Klinkert et al., 2004). However, no synthetic small-diameter vascular grafts (<6 mm) have been approved for clinical applications (Pektok et al., 2008; de Valence et al., 2012). Decellularized biological vascular grafts from xenogenic or allogenic tissues have shown great potential to serve as a new generation of tissue engineering vascular grafts (TEVG) which are composed of an extracellular matrix (ECM), sufficient collagen, elastin, and glycosaminoglycan (GAG). They can provide a friendly microenvironment due to their abundant cell signaling factors that are conducive to cell adhesion, proliferation, migration, and differentiation (Schmidt and Baier, 2000). Furthermore, the decellularized grafts can successfully match size, length, and compliance between donor and recipient vessels. However, the decellularized biological vascular grafts may have incomplete removal of cellular components or alterations in the structure of the ECM and exposure of collagen fibers. Several reasons of failure including thrombosis, immune rejection, aneurysm, intimal hyperplasia, and calcification are still challenges before the clinical application of xenogeneic small-diameter vascular grafts (Lin et al., 2018).

There are a few decellularized products on the market, such as the bovine carotid artery (Artegraft^®^), bovine mesenteric vein (ProCol^®^), and bovine ureter (SynerGraft^®^), which are used for clinical applications (Sharp et al., 2004; Das et al., 2011; Pashneh-Tala et al., 2016; Lin et al., 2018). The bovine internal mammary artery (BIMA) is used as a substitute for heterogeneous small-caliber blood vessels, but it has not undergone decellularization and properly cross-linking treatment, resulting in certain limitations in clinical application (Mitchell et al., 1993; Craig and Walker, 1994; Esposito et al., 1994). However, the BIMA also has a desired length (15–20 cm) and its diameter uniformly changes from 5 to 3 mm from the proximal to the distal end, which is conducive to clinical requirements, such as the replacement of peripheral blood vessels, the implantation of coronary artery bypass grafts, and the hemodialysis of the fistula (Catto et al., 2014; Mahara et al., 2015). Therefore, the BIMA is a suitable candidate material for the construction of small-diameter vascular grafts in tissue engineering.

The combination of different protocols, such as detergent, physical shake, and biological enzyme, was the most commonly used decellularized method (Crapo et al., 2011). Based on our previously described multistep detergent and enzymatic decellularization procedure (Lü et al., 2009), we modified a new decellularization strategy that combines sonication and perfusion, which can be used to reduce the concentration of detergent and exposure time and preserve the three-dimensional structure of the extracellular matrix. Previous studies have shown that alterations in the ECM structure, which depends greatly on the decellularization process, also lead to changes in the mechanical properties. In addition, the decellularization process can reduce the antigenicity of xenogeneic biomaterials (Böer et al., 2011), but there is still more inflammatory cell infiltration after *in vivo* implantation, and cross-linking can inhibit immune inflammatory cell infiltration and tissue degradation (Courtman et al., 2001). The physical and chemical cross-linking method can improve biomechanical properties and biocompatibility performance and reduce an inflammatory response by inhibiting the penetration of inflammatory cells or blocking part of the antigenic sites exposed by the denaturation of protein of ECM secondary conformation (Stock and Schenke-Layland, 2006). Chemically, glutaraldehyde, carbodiimide, and polyglycidyl ethers are common cross-linkers as well as physical cross-linking methods that include photooxidation and dehydrothermal methods. All of these methods mediate the formation of covalent bonds between amine groups and carboxyl groups on the collagen fibers of the bioscaffold. Although chemical cross-linkers, such as glutaraldehyde, can achieve better cross-linking effects, their residual toxicity or excessive cross-linking can cause calcification or less cellular infiltration, leading to failure (Schmidt and Baier, 2000; Parenteau-Bareil et al., 2010).

Photooxidation cross-linking has the advantages of non-toxicity, less calcification, and mild inflammation, but it does not cross-link in elastic fibers, which is also responsible for failure of vascular implantation, such as early degradation of elastic fibers, aneurysmal dilatation, and impaired vascular mechanical properties (Moore and Adams, 2001; Meuris et al., 2003). Our previous studies have shown that photooxidation cross-linking of decellularized bovine jugular vein can prevent ECM degradation and promote the regeneration of collagen, GAGs, and cell infiltration, but the elastic fibers are gradually degraded, which may lead to binding of elastin molecules’ peptide chain backbone to calcium ions, resulting in calcification. Elastic fibers are richer in the arterial walls; therefore, elastic fibers need to be further processed (Lü et al., 2010). PGG is a polyphenol antioxidant and cross-linker of elastic protein and has the function of stabilizing elastic fiber, reducing the degradation and calcification of ECM, and attenuating excess reactive oxygen species (ROS) and anti-inflammatory effects in the regulation of an inflammatory response (Fraga et al., 2010; Leopoldini et al., 2011).

Overcoming early acute thrombosis, an acute inflammation, degradation, and calcification in a decellularized small-diameter vascular xenograft is challenging. We hypothesized that a modified decellularization protocol with photooxidation and PGG cross-linking could improve the properties of biomechanical and biocompatibility; reduce inflammatory responses, degradation of elastin, calcification; and promote neointimal stabilization and remodeling. To test this hypothesis, we used BIMA as a tissue engineering vascular graft, which is based on a modified decellularization strategy that combines sonication, perfusion, and cross-linking with photooxidation and PGG in this study. In addition to quantification of DNA and ECM proteins, characterization of surface topography, biomechanical strength, fixation index, susceptibility to elastinase, cell biocompatibility *in vitro*, short-term subcutaneous transplants in rat, and abdominal aorta replacement in rabbit were performed to assess graft performance, cell migration, remodeling, and mass spectrum of neointima *in vivo*.

## Materials and Methods

**FIGURE 1:**
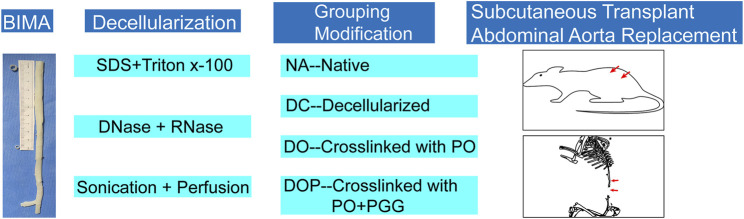
Experimental setup. After decellularization by ultrasound and perfusion, the vascular grafts were grouped, modified by cross-linking, and implanted in the animal models. PO, photooxidation; PGG, pentagalloyl glucose.

Fresh BIMA was received from a local slaughterhouse, cleaned from adherent tissues as well as residual fat, and consecutively rinsed using phosphate-buffered saline (PBS). In addition, 10-cm segments were cut from the BIMA, and the segments (n = 80) were stored at 4°C in PBS supplemented with 1% streptomycin/penicillin. The blood vessels in our laboratory are generally stored for 2 weeks in PBS containing 1% streptomycin/penicillin at 4°C. After gamma radiation sterilization at a dose of 25 KGy, the grafts can be stored in 60% alcohol for 1 year. Thereafter, the BIMA was further processed as indicated in our flow scheme (Figure 1).

### Decellularization Procedure

The decellularization procedure was based on a combination of physical, chemical, and biological methods and a system which consisted of an ultrasonic cell breaker and a peristaltic pump. The steps were as follows: first, we used a peristaltic pump (80–100 ml/min) to perfuse BIMA with 0.5% sodium dodecyl sulfate (SDS, Sigma, L3771, United States) and 0.5% Triton X-100 (Sigma, X-100, United States) for 24 h and combined with sonication treatment (20 kHz, 200w) for 12 h. Second, after sonication treatment, samples were washed with PBS solution by using the peristaltic pump for 30 min and DNAse (40 U/ml, Servicebio, G5043, China) and RNAse (0.3 mg/ml, Servicebio, G3413, China) was added in the decellularized container at 37°C water bath for 24 h. Third, after ribozyme treatment, samples were washed in the perfusion system with PBS for 48 h. Finally, the decellularized BIMAs (DC) (n = 60) were stored in sterile PBS at 4°C before further use.

### Photooxidation and PGG Cross-Linking

Decellularized BIMAs were cross-linked by using a dye-mediated photooxidation technique, according to the method of Moore et al. (1994). The experimental process refers to our previous research (Lü et al., 2010). In brief, the grafts were immersed in a chilled PBS containing glucose (pH 7.6, Osm: 680 osm) for 4 h, followed by a 2-step cross-linking process. First, the grafts were incubated in a cross-linking solution, a chilled PBS containing glucose (pH 7.6, Osm: 320 osm) that comprised 0.1% methylene blue (Sigma, M9140, United States) for 4 h at 4–20 °C in the dark. In the second step, the cross-linking solution and DC graft reacted with a 500-W broad wavelength light source and worked for 48 h at 0–15 °C with 10 ml/min air sparged through the stirred reaction solution. The inner and outer walls of the grafts were treated by photooxidation for 24 h, and the reaction solution was changed every 24 h. After cross-linking, the DC with photooxidation cross-linking grafts (DO, n = 20) was washed in sterile PBS and stored in sterilized PBS at 4°C for further study.

The DO grafts were cross-linked with different concentrations (0.1%/0.25%/0.5%) of sterile PGG (Biopurify, 14937-32-7, China) in 50 mM phosphate buffer of pH 5.5 containing 20% isopropanol for 24 h (Pennel et al., 2014), and then the PGG-treated DO-BIMA (DOP, n = 20) was washed three times with PBS. All the grafts were sterilized by 25 KGy gamma irradiations and stored in 60% alcohol at 4°C for further use.

### DNA Quantification and Electrophoresis

The total DNA amount of native BIMA (NA) or DC-BIMA grafts was quantified by using a DNA detection kit (TIANamp Genomic DNA kit) for grafts. We then prepared an agarose gel, followed by making an electrophoresis gel membrane, and finally added a marker and DNA samples, respectively. The electrophoresis parameters were 100 V of current for a duration between 30 and 45 min. Then the electrophoresis gel membrane was exposed under UV light and scanned the picture.

### Histological Analysis

The samples were fixed in 4% formaldehyde solution for 24 h, followed by gradient dehydration in alcohol. Then paraffin infiltration and embedding were performed, and slides of 5 μm each were cut out. Then the slides were stained by hematoxylin and eosin (H/E, Servicebio, G1005, China) stain for general morphology and cell nuclei, Masson’s trichrome (Servicebio, G1006, China) stain for collagen demonstration, elastic van Gieson (EVG, Servicebio, GP1035, China) stain for elastin demonstration, and immunohistochemistry (IHC) stain for identification of the α-gal antigen.

### Mechanical Testing

The four groups of NA/DC/DO/DOP-BIMA were cut into short conduits with a diameter of 4 mm and a length of 10 mm (n = 6). The grafts were used to measure the radial mechanical properties, burst pressure, and suture tension. An INSTRON instrument, which measures radial mechanical properties, was used. In addition, 2-cm length of graft (n = 6) was used for a suture retention test, performed by placing a 6-0 prolene suture 2 mm from the end of the graft and pulled to failure on an INSTRON tensile testing system. The burst pressure was measured by delivering PBS from a 50-ml syringe *via* a control valve. The diametrical compliance (C) was calculated by using equations published by Hamilton’s group (Tai et al., 1999). In brief, the diameters were recorded between pressure of 120 and 80 mm Hg by slowly injecting PBS into each group of vascular grafts (n = 6).

### Fixation Index Determination

The ninhydrin (NHN) assay can be used to reflect the degree of tissue cross-linking by the fixation index (FI). The FI was calculated by using equations published by Hsu’s group (Sung et al., 1996). In brief, after being fixed, all samples were lyophilized and weighted. Then tissues were put into NHN solution (Aladdin, N105629, China) and heated to boil for 20 min. Optical absorbance of the solution was detected by a microplate absorbance reader at 570 nm.

### Scanning Electron Microscopy

For scanning electron microscopy (SEM), the NA/DC/DO/DOP-BIMA samples were fixed and dried by a graded ethanol series and then freeze-dried for dehydration. Dried samples were sputter coated with 20 nm of gold and analyzed using a FEI Nova NanoSEM (FEI Electron Optics B.V, Czech). The number of pores, ratio of the pore area, and diameter of a fiber were calculated by Image-Pro Plus 6.0 analysis software.

### Elastin Degradation Experiments

To test the elastin degradation ability of NA/DC/DO/DOP-BIMA, different grafts were freeze-dried and weighed. Elastase (20 IU/ml, Biomatik, A4126, China) was added and the grafts were incubated at 37°C for 24 h; then the grafts were freeze-dried and weighed. Finally, the degradation rate of each group’s materials was calculated.

### Cell Biocompatibility

#### Cytotoxicity Assessment

The human umbilical vein endothelial cell line (EAhy926) was cultured in a high glucose Dulbecco’s modified Eagle mediumwith 10% fetal bovine serum (DMEM/10%FBS, Servicebio, G4510, G8001, China). A sample size of 1 × 1 cm^2^ was cut from BIMA grafts and cultured in DMEM/10%FBS at 37°C for 24 h at a density of 2.5 ml/cm^2^. The culture media (leach liquor) were collected and preserved. The media were replaced with leach liquor from the scaffold cultures diluted with DMEM/10%FBS at a ratio of 1:2. The cells were cultured for a further 1, 3, and 5 days at 37°C. A negative control was prepared using DMEM/10%FBS alone. The mitochondrial metabolic (MTT, Servicebio, G4104, China) assay was applied in assessing the cell growth on the scaffold. The optical density at 570 nm was determined using a microplate reader. The cytotoxicity of each protocol was evaluated by calculating the relative growth rate (RGR) to determine the proliferation index (Xu et al., 2017). RGR = (mean OD for each group)/(mean OD of the negative control) × 100%.

#### Cell Viability Assay

Samples of the four groups were cut into 5 × 5 mm pieces, washed with sterile PBS for 20 min, soaked in DMEM/10%FBS for 24 h, and then placed in a 48-well plate. A total of 5 × 10^3^ cells were seeded on each sample. After 30 min, 500 μl of DMEM/10%FBS was added to each well and cultured for 7 days. The culture medium was changed every 2 days. The pieces of vascular grafts were transferred to clean wells and washed with a PBS solution, and 100 μl of 10 μmol/ml 6-CDCFDA (carboxy-2′,7′-dichlorofluorescein diacetate, Yuhengbio, C4096, China) live cell fluorescent dye was added to each well and incubated for 30 min in incubators. Cells were visualized by using an inverted fluorescence microscope. Then the pieces were fixed and prepared for SEM. In addition, we used immunofluorescence staining of F-actin to show the morphology of cells with phalloidin (Servicebio, G1028, China) and acquired images using a laser scanning confocal microscope (LSCM, LSM780, Zeiss, Germany).

### *In Vivo* Subcutaneous Implantation in Rat and Abdominal Aorta Replacement in Rabbit

#### Subcutaneous Implantation

All animal experiments were approved by the Animal Research and Ethics Committee of The Second Xiangya Hospital of Central South University (CSU) and performed by the Department of Cardiac Surgery of The Second Xiangya Hospital. Host cell infiltration and immune inflammation responses against the different groups of NA/DC/DO/DOP BIMA (n = 4) were evaluated in rat subcutaneous implantation models. In this regard, about 4-week-old Sprague–Dawley rats (SD) with mean weight of 140 g were selected and anesthetized by sodium pentobarbital (30 mg/kg), and the vascular grafts with the size of 1 × 1 cm^2^ were implanted on each side of the subject rats. A 2-0 Mersilk suture was used to suture the skin of rats. Finally, the samples were removed to undergo histological examination after 28 days by using H/E staining. IHC staining was performed using anti-mouse CD3 (1:400, Servicebio, GB11014, China) and CD68 (1:200, Servicebio, GB11067,China) primary antibodies.

#### Abdominal Aorta Replacement

Grafts of three groups (DC/DO/DOP, n = 5) were evaluated in a total of 15 rabbits. The length of the grafts was 2 cm, and the diameter was 3.5 mm. Animals were anesthetized and sedated with 30 mg/kg sodium pentobarbital. Following a left paraspinal incision, the aorta was dissected free of surrounding tissues and the inferior vena cava. Intravenous heparin (1 mg/kg) was administered before implantation and grafts were implanted into infrarenal aorta by end-to-end anastomoses using 7-0 prolene sutures. All grafts were implanted for 4 weeks to compare the progression of remodeling of neointima, inflammation degradation of elastin, and calcification. Prior to the animals being euthanized, abdominal computed tomography angiography (CTA) was performed at 4th week, and the graft diameters were measured to evaluate the presence or absence of stenosis. After 4 weeks, grafts were then excised and washed with saline. The samples were analyzed histologically using the HE staining, Masson’s trichrome staining, and von Kossa (Servicebio, G1043,China). The EVG staining for elastin and quantification of elastin’s relative content from the histological images was performed by Image-Pro Plus 6.0 analysis software. The content of elastin in implanted grafts was compared with that in pre-implanted grafts to calculate the preservation ratio of elastin. The identification of different types of collagen fibers and content ratios in the neointima was determined by Picro-Sirius Red staining (Abcam, ab246832, United Kingdom). Studies have used the differences between the appearances of different collagen fibers under polarized light to differentiate between different types of collagen fibers. The color of type I collagen fibers is yellow/orange birefringence and type III collagen fibers is green birefringence. Using Image-Pro Plus 6.0 analysis software, the pixel area of type I collagen and the pixel area of type III collagen in the neointimal area of each group was measured uniformly with the pixel area as the standard unit; the type I and type III collagen pixel area ratios were calculated. IHC or immunofluorescence (IF) identification of endothelial cells was performed with antibodies to CD31 (1:200, Abcam, ab9498, United Kingdom), CD68 for macrophages (1:200, Servicebio, GB14043, China), CD206 for M2 macrophages (1:1,000, Abcam, ab254471, United Kingdom), alpha-smooth muscle cell actin (1:200, Gene Tex, GTX18147, United States) for fibroblast, calponin (1:500, Abcam, ab700, United Kingdom) for smooth muscle cell, and vimentin (1:200, Abcam, Ab8978, United Kingdom) for mesenchymal cell. The exact protocol is described in the Supplementary Material.

### Mass Spectrum

The sample was rinsed and wiped dry using a dust-free paper. In total, 8 M urea +100 mM Tris-HCl (pH 7.2) lysate (with protease inhibitor) was added to solubilize protein. The protein concentration was determined by using the BCA method. A total of 500 μg protein was extracted from each group of samples; then mass spectroscopy-grade trypsin was added with at a ratio of 1:50 and digested overnight at 37°C. The peptide-containing solution was freeze-dried, and the peptide was dissolved for a mass spectrometry analysis. The Nono LC. LTQ Oribtrap MS was used for the mass spectrometry analysis. An electrospray ion source using a positive ion mode detection with a spray voltage of 2.4 kV at an ion source temperature of 350°C was used. The first-level FT scan was at the resolution of 60000 FWHM; the second-level IT scan was within the range of 280–1,500, in the TOP 20 mode, at the resolution of 7500 FWHM with a collision energy at 35%. The software Protome Discovery 2.0 (Thermo Fisher) was used to search the MS RAW files in the Uniprot TrEMBL database. Search database parameter settings are as follows: the species is rabbit; the cleavage enzyme is trypsin; the maximum number of missed cleavages is 1; the error of primary precursor ion is 10 ppm; and the error of secondary daughter ion is 0.5 Da. A false discovery rate of 0.01 was used and two biological replicates were analyzed. Proteins were stated identified if at least three unique peptides were detected.

### Statistical Analysis

The results of quantitative studies were expressed as the median and the interquartile range. For analysis of two groups with unpaired samples, the Mann–Whitney *U* test was used. For analysis of three or more groups, the Kruskal–Wallis test with Tukey’s post hoc test was used and analyzed using a statistical analysis software IMB SPSS Statistics 22.0 (IBM, United States), and graphs were generated using GraphPad Prism 8 (GraphPad, United States). *p*-values ≤ 0.05 were considered to be statistically significant.

## Results

### Decellularized BIMA Preparation and Characterization

**FIGURE 2:**
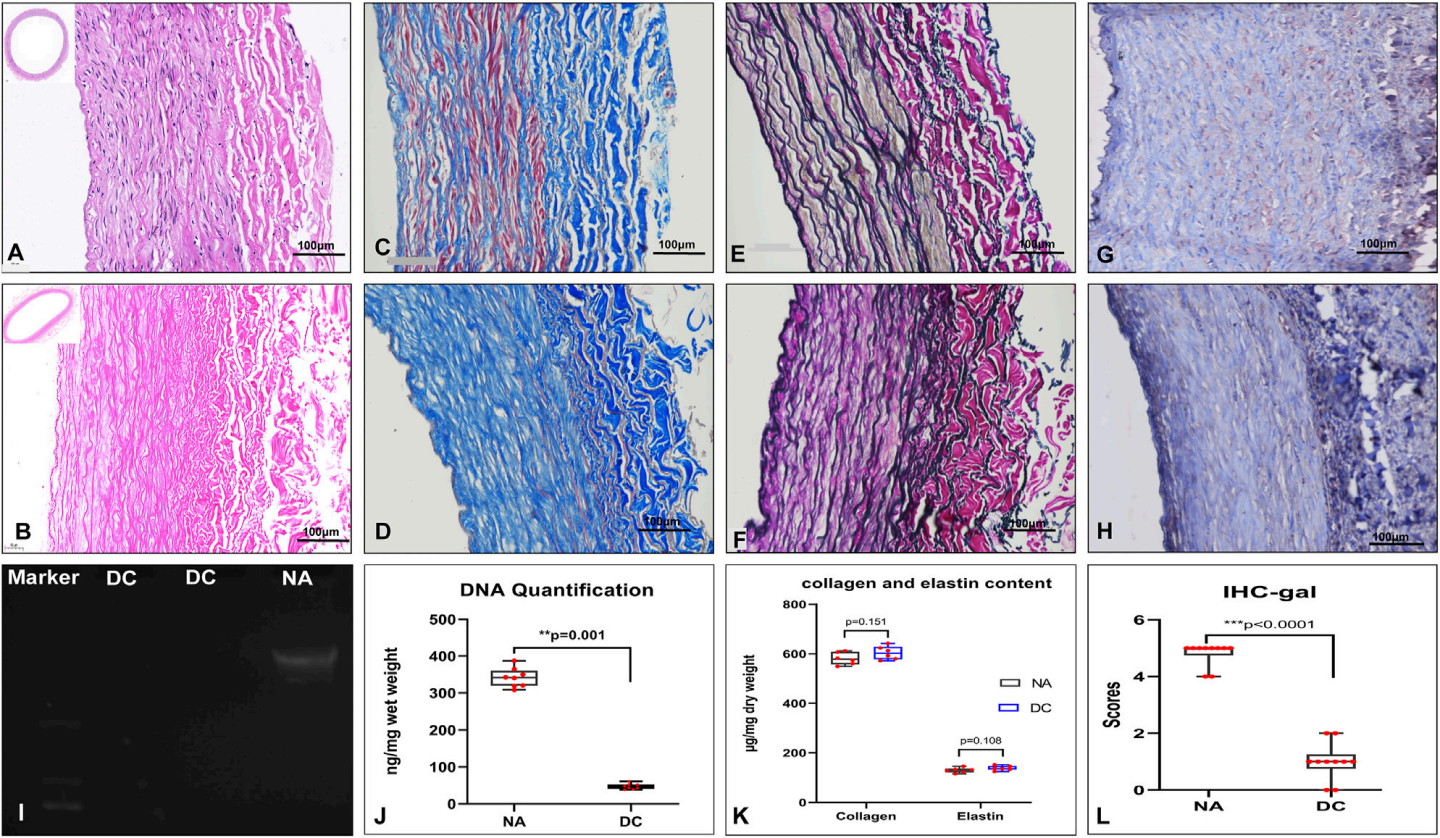
Characteristics of the bovine internal mammary artery (BIMA) before **(A,C,E,G)** and after **(B,D,F,H)** acellularization. H&E staining **(A,B)**, Masson’s trichrome staining **(C,D)**, van Gieson staining **(E,F)**, and immunohistochemistry of α-gal **(G,H)**. **(I)** DNA electrophoresis showed no macromolecular bands of the decellularized bovine internal mammary artery (BIMA). **(J)** DNA quantification of native and decellularized BIMA (Mann–Whitney *U* test). **(K)** Content of collagen and elastin was insignificant between the NA and DC groups (Mann–Whitney *U* test). **(L)** Comparison of α-gal antigen content (IHC scores) of native and decellularized BIMA (Mann–Whitney *U* test). NA, native BIMA; DC, decellularized BIMA.

The decellularized BIMA with a diameter of 3–6 mm and a length of 10–15 cm were obtained. The acelluar BIMA showed optimal three-dimensional structure of the extracellular matrix by HE, Masson, and EVG staining (Figure 2). The comparison of expression levels of α-gal (IHC scores) in NA and DC groups showed that α-gal antigen were significantly reduced in decellularized grafts (Figure 2L). DNA electrophoresis (Figure 2I) showed no macromolecular bands, and the DNA content (Figure 2J) was significantly reduced in decellularized grafts (Figure 2). The content of collagen and elastin was not significantly different between the NA and DC groups (Figure 2K). The BIMA showed a complete internal elastin lamina membrane, thin media, which contained collagen and elastic fibers, and a relatively loose adventitia.

### Mechanical Properties of Cross-Linked BIMA

**FIGURE 3:**
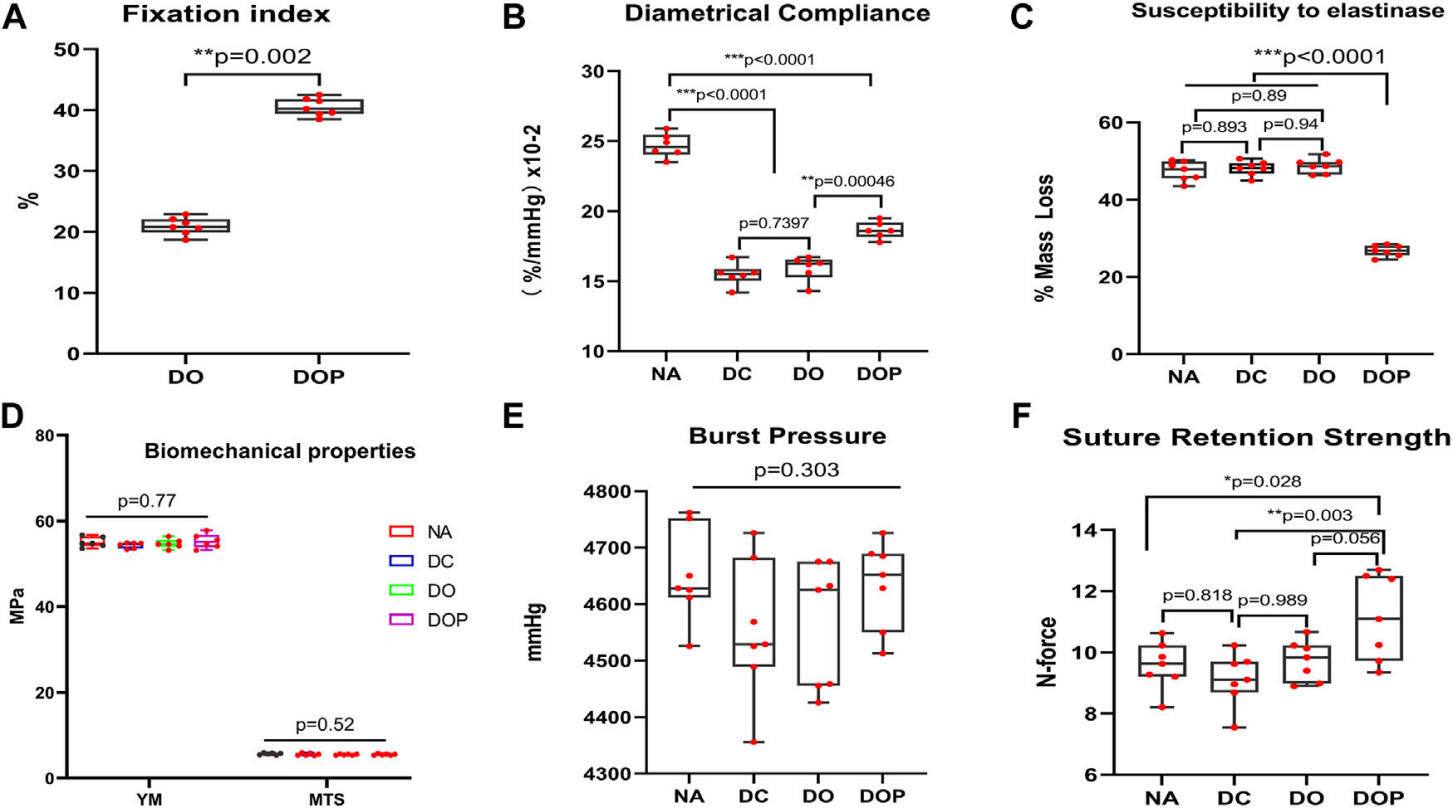
Bovine internal mammary artery’s (BIMA) property before and after cross-linking. **(A)** Fixation index of the DOP group was significantly increased compared to the DO group (Mann–Whitney *U* test). **(B)** Diametrical compliance significantly decreased in the DC, DO, and DOP groups, but it was significantly increased in DOP groups compared with the DC and DO groups. **(C)** Resistance to elastinase in DOP groups was significantly higher than that in the NA, DC, and DO groups. **(D,E)** Difference of the four groups’ burst pressure and radial mechanical was insignificant. **(F)** Suture retention strength of the DOP group significantly increased compared to the NA and DC groups. For B, C, D, E, and F, box and whiskers, minimum to maximum, shows all points. The Kruskal–Wallis test with Tukey’s post hoc test. NA, native BIMA, DC, decellularized BIMA; DO, photooxidation cross-linked BIMA; DOP, photooxidation and PGG cross-linked BIMA; YM, Young’s modulus; MTS, maximum tensile stress.

To test the effect of cross-linking on the biomechanical properties of vascular scaffolds, the ninhydrin experiment, the compliance, and the resistance to elastase were assayed. The aforementioned three experiments were tested in the DOP groups (0.1%, 0.25%, and 0.5%), and the results indicated that moderate cross-linking of PGG improved the fixation index and resistance to elastase, but excessive cross-linking also led to reduced vascular compliance. Therefore, 0.25% PGG was chosen as our cross-linked concentrations. (The comparison data of different concentrations of DOP group are shown in the Supplementary Material.) The ninhydrin experiment results showed that the cross-linking degree of the DO and DOP groups was approximately 20% and 40%, respectively (Figure 3A). The compliance was approximately 24%, 14%, 15%, and 17% in the NA, DC, DO, and DOP groups, respectively (Figure 3B). The compliance of the DOP group was lower than that of the NA group but significantly higher than that of DC and DO groups. The elastin degradation test showed that the DOP group had the lowest weight compared with the other three groups (Figure 3C).

After comparing the NA, DC, DO, and DOP groups’ radial mechanical properties, we found that there was no significant difference in Young’s modulus, maximum tensile stress, and burst pressure among the four groups (*p* > 0.05, as shown in Figure 3D and Figure 3E), but in the suture retention strength, the DOP group was significantly higher than NA and DC, and there was an insignificant difference from the DO group (Figure 3F).

### SEM and Staining

**FIGURE 4:**
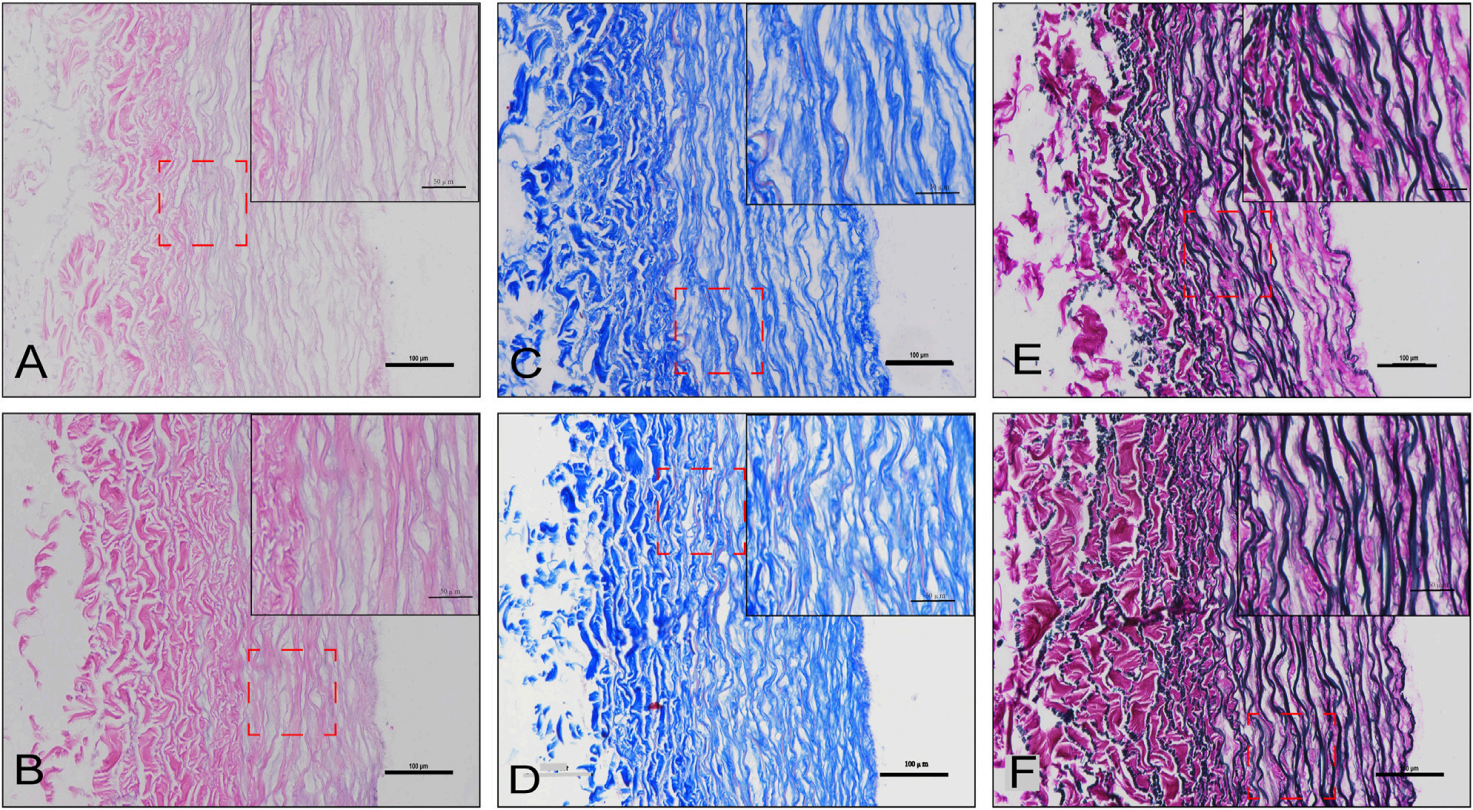
Histological staining of the DO and DOP groups. H&E staining **(A,B)**, Masson’s trichrome staining **(C,D)**, and van Gieson staining **(E,F)** of the DO **(A,C,E)** and DOP **(B,D,F)** groups.

**FIGURE 5:**
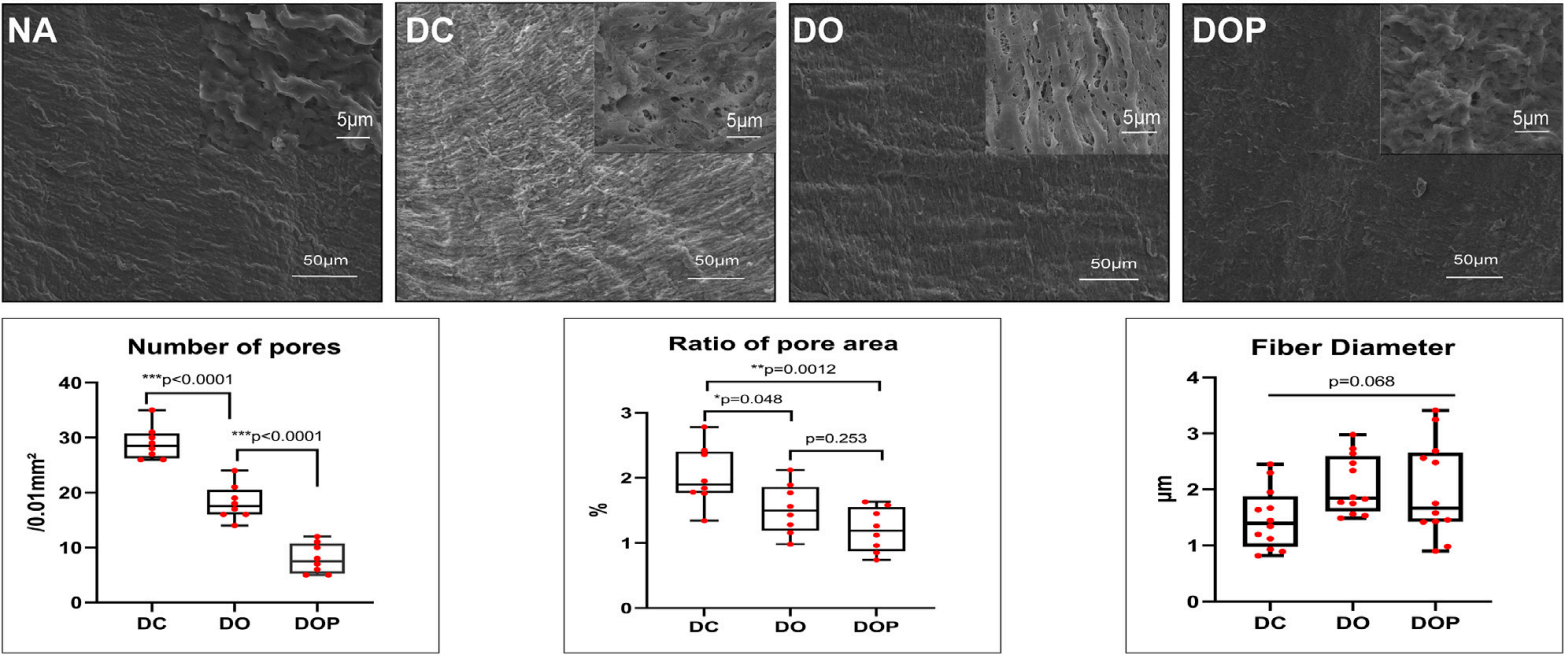
Morphology assessed by SEM and semi-quantitative results of pore number, area, and mean fiber diameter in the groups. The number of pores and the ratio of the pore area in the DOP group were significantly less than those in the DO and DOP groups. The difference of fiber diameter was insignificant in the groups. Box and whiskers, min to max, show all points; The Kruskal–Wallis test with Tukey’s post hoc test. NA, native BIMA; DC, decellularized BIMA; DO, photooxidation cross-linked BIMA; DOP, photooxidation and PGG cross-linked BIMA.

To observe the structure and morphology of blood vessels after cross-linking, HE/MASSON/EVG staining were performed in DO and DOP groups (Figure 4). From the SEM results, the NA group showed a tight fiber connection without obvious pores. The SEM images of the same area of DC/DO/DOP groups were selected, and then the number of pores per unit area and ratio of pore area were counted and analyzed which demonstrated that there were significant differences among the groups. The DOP group showed that the number of pores and the ratio of pore areas were less than that of other groups (Figure 5).

### Cell Biocompatibility

**FIGURE 6:**
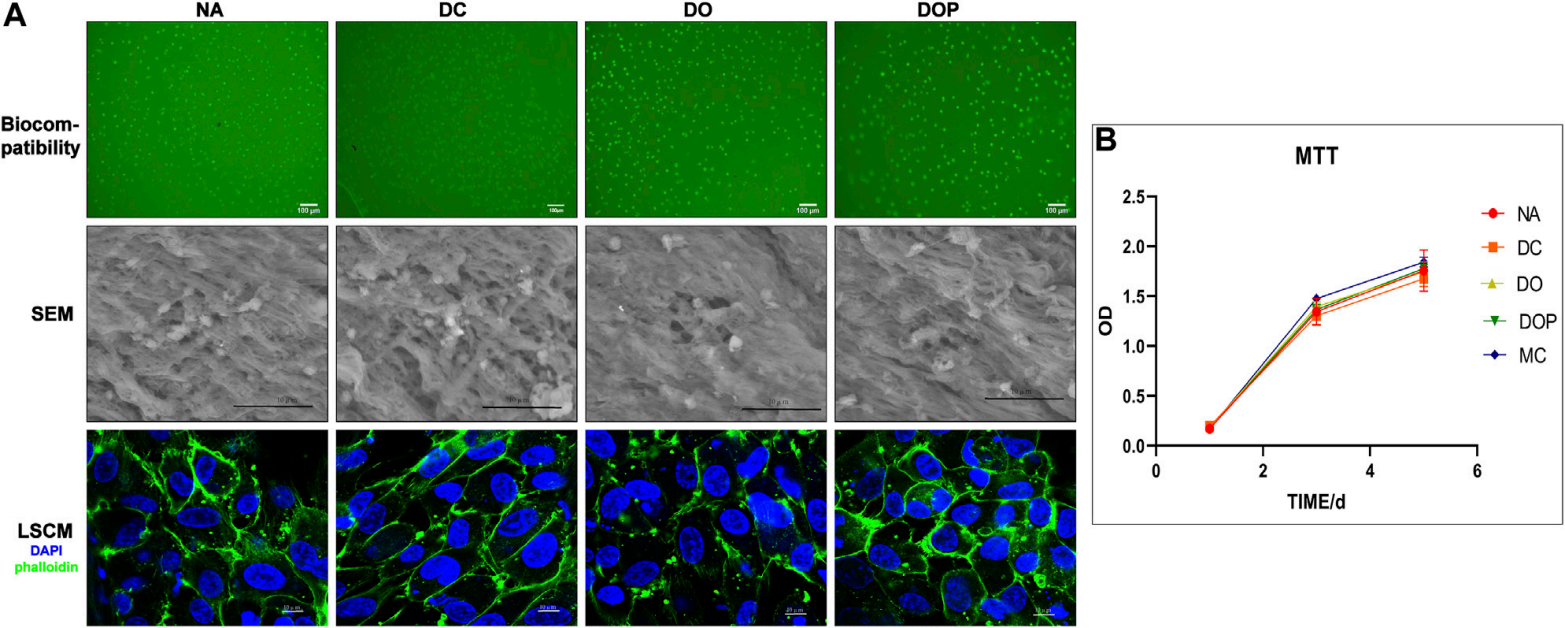
Cell biocompatibility of the NA, DC, DO, and DOP groups are shown. **(A)** Human umbilical vein endothelial cells adhered and spread on the materials after 7 days, and cell adhesion and morphology was assessed by 6-CDCFDA live cell fluorescent staining, scanning electron microscopy (SEM), and the immunofluorescence staining of F-actins for the cell shape (DAPI: blue; phalloidin: green). **(B)** Cytotoxicities were determined by the MTT assay and showed no significance in the relative growth ratio between the experiment groups and control groups (Kruskal–Wallis test with Tukey’s post hoc test, *p* > 0.05). NA, native BIMA; DC, decellularized BIMA; DO, photooxidation cross-linked BIMA; DOP, photooxidation and PGG cross-linked BIMA.

The cytotoxicity of the four groups’ scaffolds was determined by using the MTT assay. As shown in Figure 6, the relative growth ratios (RGRs) of HUVECs were grown in the presence of leach liquor from the scaffolds at concentrations after 1, 3, and 5 days of culture were evaluated. On Day 1, no apparent differences were observed in the RGRs between the four groups; on Day 3 and Day 5, the RGRs in the four groups reached level 1 and showed an insignificant difference between different groups. For the cell viability assay of the DC, DO, and DOP groups, the cells adhered and spread on the materials. The live cell 6-CDCFDA fluorescent staining and results of SEM and IHC-CD31 confirmed the reseeded cells’ viability, and there were no significant difference in the four groups. The F-actins’ immunofluorescence staining of phalloidin showed that the cells adhered to the luminal surface of the vessels in a cobblestone shape. (The results of IHC show in the Supplementary Material.)

### Host Cell Infiltration and Inflammation of the Grafts *In Vivo*

**FIGURE 7:**
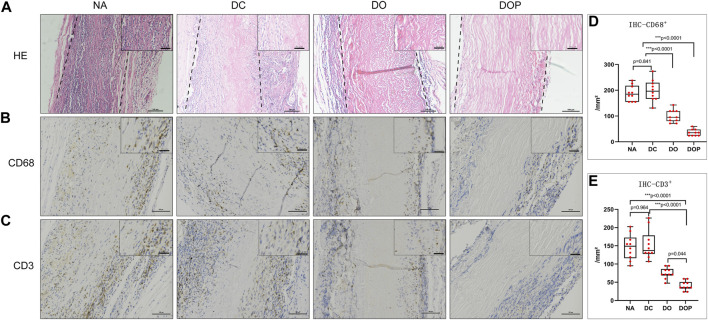
Histological staining of four groups’ grafts implanted into the subcutaneous transplants of rats for 4 weeks. **(A)** Histological staining (H&E). The dotted line depicts the edge of the implanted graft; **(B)** immunohistochemical (IHC) staining for macrophages (CD68); **(C)** IHC staining for T lymphocyte (CD3); **(D,E)** quantitative analysis of the number of CD68^+^ and CD3^+^ cells in the four groups. The number of positive cells in the NA group and DC group was significantly higher than the DO and DOP groups, and the number of positive cells in the DOP group is significantly reduced (box and whiskers, min to max, show all points; Kruskal–Wallis test with Tukey’s post hoc test). NA, native BIMA; DC, decellularized BIMA; DO, photooxidation cross-linked BIMA; DOP, photooxidation and PGG cross-linked BIMA.

Before the end of the study, the rats did not receive any drugs and antibiotics. After 28 days, no reactions, including local and systemic infections as well as infections of the implanted scaffolds in animal models at the surgical sites, were observed. The four groups’ HE stain examination showed that in the NA group, many cells were infiltrated in the graft and parts of the internal and adventitia membranes were degraded (Figure 7). In the DC group, many cells were infiltrated in the graft, and the internal and adventitia membranes were partially degraded. In the DO group, a small cell amount in the graft was infiltrated; the blood vessel was not degraded. In the DOP group, there was almost no cell infiltration in the graft, and the structure of the graft was basically intact. The IHC results showed the presence of more CD68^+^ and CD3^+^ cells in the NA and DC groups (*p*<0.01), distributed in the vascular wall and the fibrous capsule. Compared with the DOP group, there were more CD68^+^ and CD3^+^ cells in the DO group that infiltrated in the superficial vascular wall and the fibrous capsule, while the positive cells in the DOP group only existed in the fibrous capsule (Figure 7).

### *In Vivo* Performance of Abdominal Aortic Implantation

**FIGURE 8:**
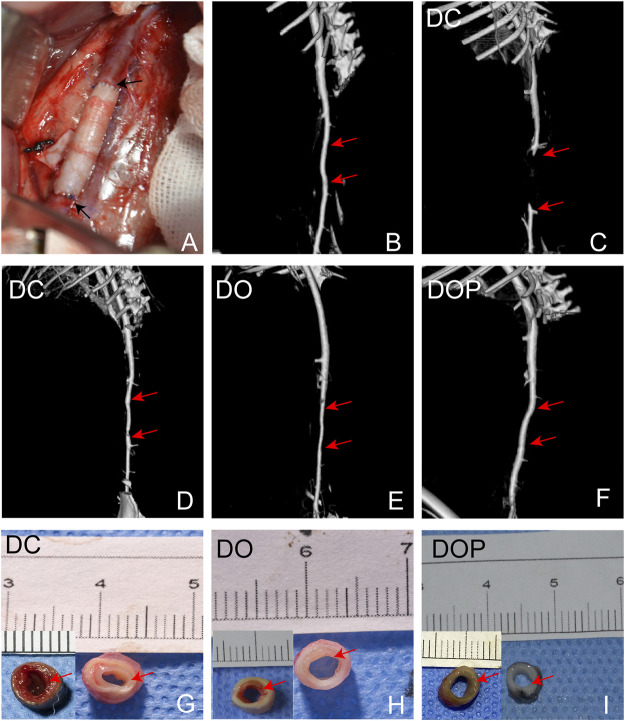
**(A)** Macroscopic images of the rabbit infrarenal abdominal aortic transplantation model. **(B)** CTA results on the first day after operation. **(C,D)** CTA results of occlusion and stenosis after 1 month of vascular implantation in the DC group. **(E)** CTA results of stenosis after 1 month of vascular implantation in the DO group. **(F)** CTA results of DOP group after 1 month of vascular implantation. Red arrows depict the anastomoses; the mid-section gross images of grafts implanted for 1 month in the DC **(G)**, DO **(H)**, and DOP **(I)** groups. Red arrows depict the neointima. DC, decellularized BIMA; DO, photooxidation cross-linked BIMA; DOP, photooxidation and PGG cross-linked BIMA.

**FIGURE 9:**
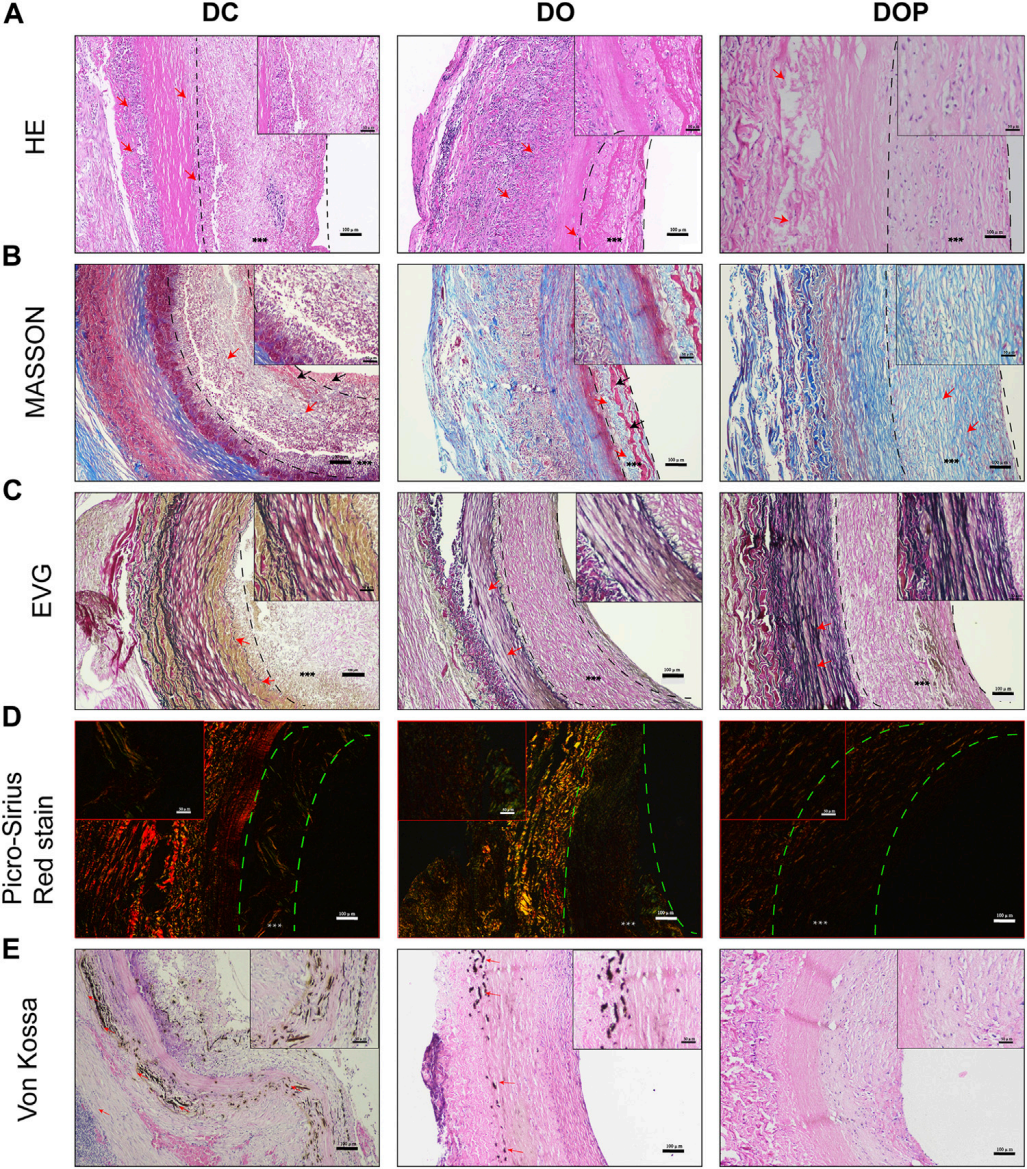
Histological staining of three groups’ grafts implanted into the abdominal aorta of rabbits for 1 month. **(A)** Histological staining. The red arrows depict cell infiltration in the basement membrane and adventitia of grafts in the DC and DO groups. In DOP group, a few cells infiltrated the adventitia of graft. **(B)** Masson’s trichrome staining. The red arrows depict the regenerated collagen fibers and structural morphology of neointima in each group. In DC and DO groups, the black arrows depict the fibrin of neointima. **(C)** Elastin van Gieson staining. The red arrows depict the structural morphology of elastin in each group of scaffolds. Elastin appeared fragmented and degraded in the DC and DO groups. **(D)** Picro-Sirius Red staining. The color of type Ⅰ collagen fibers is yellow/orange birefringence and that of type Ⅲ collagen fibers is green birefringence. **(E)** von Kossa staining: the red arrows depict the calcification. There was no calcium deposition in the DOP group. Scale bars are 100 μm. The left dotted line indicates location of basement membrane and the right dotted line indicates location of vascular graft luminal surface. *** depict the neointima. DC, decellularized BIMA; DO, photooxidation cross-linked BIMA; DOP, photooxidation and PGG cross-linked BIMA.

New Zealand white rabbits were used for abdominal aorta transplantation. All rabbits survived surgery without complications, such as paraplegia and infections. CTA results showed that the patency in the DC group was 60% at 4w and that in the DO and DOP groups were 100%. In the DC group, there were two cases of occlusion due to thrombosis. In the DO group, there were two cases with stenosis in the middle of the graft or anastomotic site (stenosis degree >30%) and two cases in the DC group (Table 1). Grossly, connective tissue adhered to the grafts’ abluminal surface explanted on the fourth week and the grafts’ luminal surface appeared glossy in the DO and DOP groups (Figure 8). The results of HE, Masson trichrome, EVG, and Picro-Sirius Red staining showed that there were a large number of cell infiltrations in the adventitia and deposition of new collagen fibers in the neointima in the three groups. In the DOP group, the new collagen fibers were arranged orderly, and the structure was similar to that of natural blood vessels; the preservation rate of elastic fibers of vascular wall in the DOP group was higher than that in the other two groups. (The comparison data of preservation rates of elastin are in the Supplementary Material.) von Kossa staining showed local calcium deposition in both the DC and DO groups but no calcium deposition in the DOP group (Figure 9).

**TABLE 1:** Summary for abdominal aorta replacement in rabbit.

	4W	Occlude	Stenosis	Patency (%)	Survival at 4W (%)
DC	5	2	2	60	100
DO	5	0	2	100	100
DOP	5	0	0	100	100

**FIGURE 10:**
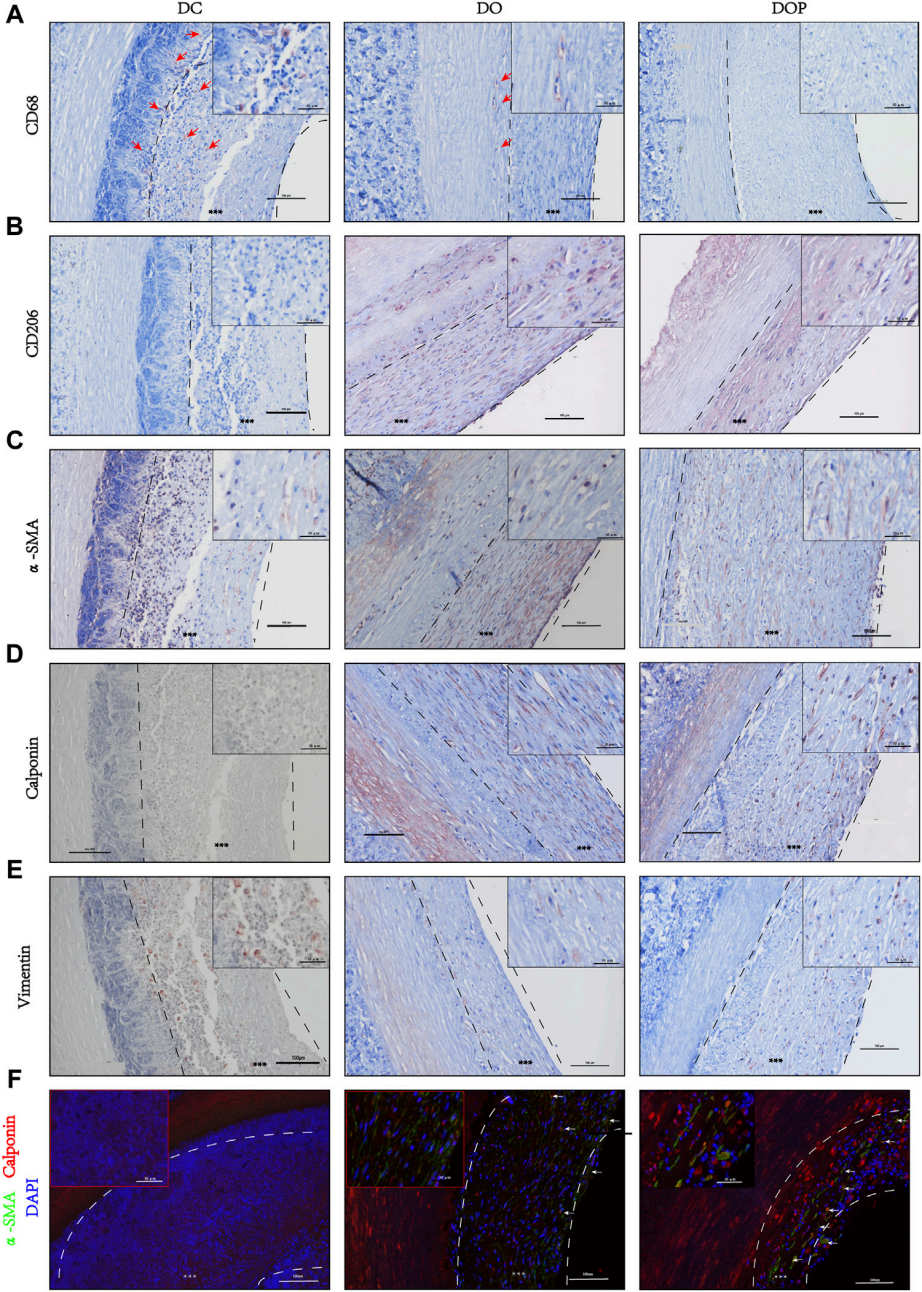
Cell histological staining of three groups’ grafts implanted into the abdominal aorta of rabbits for 1 month. **(A)** Immunohistochemical (IHC) staining for macrophages (CD68). Red arrows depict the positive cells. **(B)** IHC staining for M2 Macrophages (CD206). **(C)** IHC staining for fibroblast (α-SMA). **(D)** IHC staining for smooth muscle cell (calponin). **(E)** IHC staining for mesenchymal cell (vimentin). **(F)** Double immunofluorescence staining, α-SMA (green), calponin (red), and DAPI (blue). White arrows depict the double positive cells. Scale bars are 100 μm. The left dotted line indicates location of the basement membrane, and the right dotted line indicates location of vascular graft luminal surface. *** depict the neointima. DC, decellularized BIMA; DO, photooxidation cross-linked BIMA; DOP, photooxidation and PGG cross-linked BIMA. α-SMA, alpha-smooth muscle actin. DAPI, 4′,6-diamidino-2-phenylindole.

Results of the IHC and IF showed that α-SMA^+^ cells and vimentin^+^ cells were distributed in the neointima of all the groups. In the DOP group, there were no CD68 + cells in the vascular grafts, which could be detected in the DC and DO groups. (The comparison data of CD68^+^ cells are in the Supplementary Material.) The CD206^+^ cells and calponin+/α-SMA + double positive cells were detected in the neointima of the DO and DOP groups. However, there was no CD31 positive cell adhesion in the three groups (Figure 10).

### Mass Spectrum of Neointima

**FIGURE 11:**
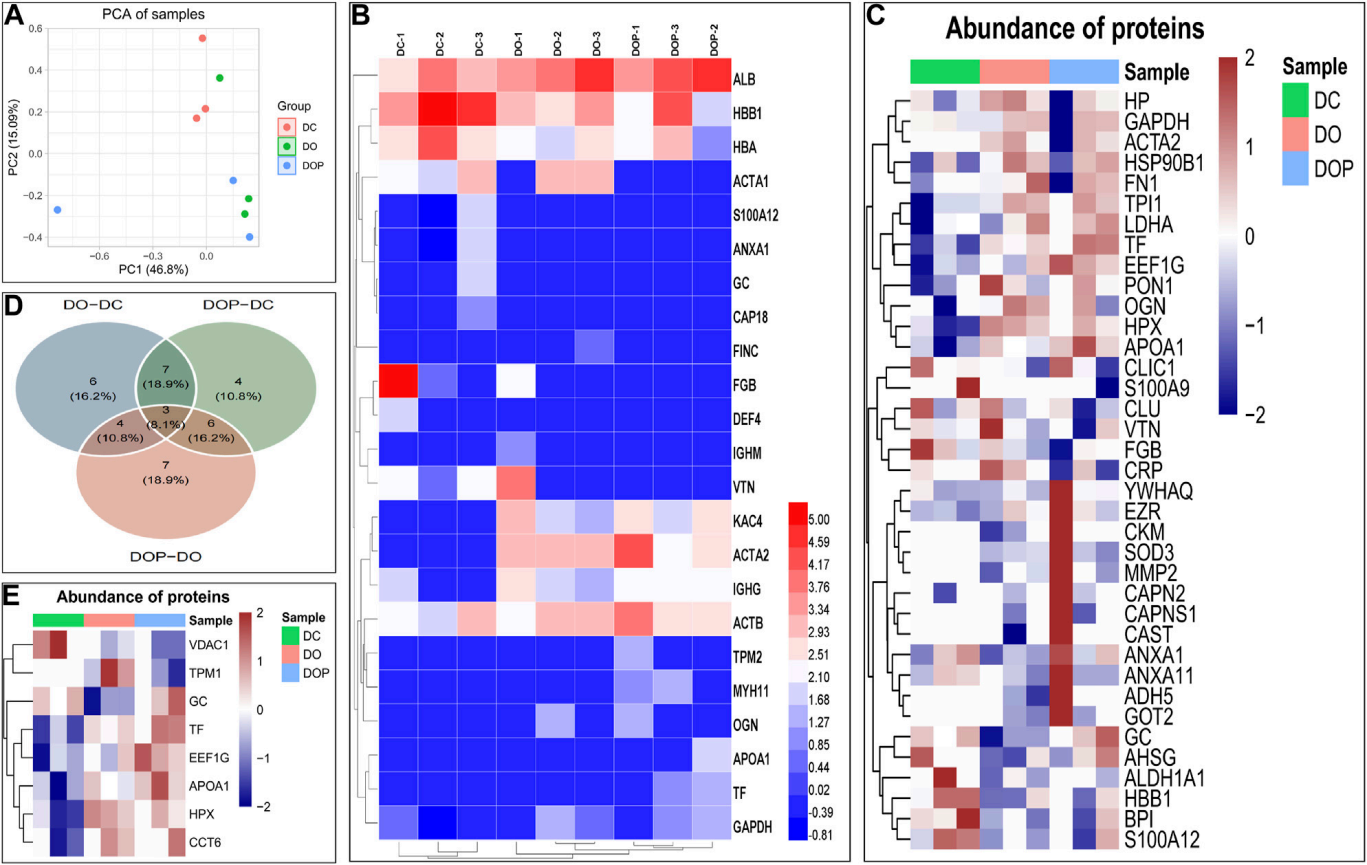
To identify the composition of the neointima, the different groups’ samples by an Orbitrap mass spectrometer using a label-free quantification method. The principal component analysis shows the relative distance between the experimental groups **(A)**. Heatmap showed that the major proteins/peptides whose abundance accounted for the top 70% of samples in the three groups **(B)**. Heatmap and Venn diagram demonstrated the proteins with the top 20 differential fold change among the three groups. The Limma package in the R language was used for difference analysis. **(C,D)** Heatmap demonstrated differential proteins with *p* < 0.05 in the three groups **(E)**. DC, decellularized BIMA; DO, photooxidation cross-linked BIMA; DOP, photooxidation and PGG cross-linked BIMA.

To identify the composition of the neointima, the different groups’ samples were digested using trypsin and analyzed by Orbitrap mass spectroscopy using a label-free quantification method. The possible components of neointima in each group were analyzed. The principal component analysis showed the relative distance between the experimental groups (Figure 11A). The results of the mass spectrometry analysis showed that the major categories whose protein abundance accounted for the top 70% of samples in the three groups were plasma protein components (albumin, hemoglobin, fibrinogen, immunoglobulin, transferrin, etc.), cytoskeletal protein components (actin–aortic smooth muscle, actin–alpha skeletal muscle, tropomyosin alpha-1 chain, etc.), glycoproteins, and secreted factors (glyceraldehyde-3-phosphate dehydrogenase, protein S100-A12, vitamin D-binding protein, etc.) (Figure 11B). We analyzed differential expression among the three groups, but no statistically significant differential proteins were found (FDR-adjusted *p* < 0.05). The heatmap and Venn diagram demonstrated the proteins with the top 20 differential fold change among the three groups (Figures 11C, D). The heatmap demonstrated differential proteins with *p* < 0.05 in the three groups (Figures 11E). GO annotations were not analyzed due to the low number of identifications of differential proteins, and FDR-adjusted *p* > 0.05. (The data of proteins/peptides are shown in the Supplementary Material.)

## Discussion

Although the artificial vascular graft of synthetic materials research and its clinical application have been in use for a long time, its complications, such as intimal hyperplasia, thrombosis, chronic inflammation, and slow tissue regeneration, are still the main reasons that limit its application in the field of small-diameter vascular grafts (Schneider et al., 2018; Schneider et al., 2020).

We described for the first time the use of decellularized BIMA in a rabbit abdominal aortic replacement model. BIMA has optimal biomechanical properties. In our study, its burst pressure was as high as 4,700 mmHg, and the suture retention reached approximately 9.0 N, which fully met the needs of human arterial blood pressure and had good durability. The results showed that the decellularization procedure combined with physical, chemical, and biological enzymes can remove cellular components and antigens of α-gal and retain the three-dimensional structure of the ECM. In our research, the decellularization results proved that the physical method of ultrasound combined with perfusion can efficiently remove cell components, and the reaction time and concentration of SDS and Triton X-100 can be reduced. The decellularization procedure was shortened to 3–4 days, which minimized the damage of detergent to ECM structure’s integrity. The DNA was almost completely removed, and electrophoresis of DNA showed no large molecular bands. The histological HE staining also indicated the cellular components’ removal. Additionally, the grafts were removed of most of the α-gal antigen and reduced of the acute natural anti-gal antibodies that mediated rejection of the xenografts (Kasimir et al., 2005; Daly et al., 2009). The Masson and EVG staining showed that the decellularized BIMA had optimal extracellular matrix morphology and a fiber structure with no obvious breakage of collagen and elastic fibers. The decellularization procedure’s short-term and effectivity could be attributed to the sonication cavitation effects and perfusion, which could break the cells and facilitate the detergent penetration through the tissue (Azhim et al., 2014; Schneider et al., 2016).

The decellularization process will cause a damage to the ECM and expose the laminin of the basement membrane and collagen structure, leading to the degradation of mechanical properties and the exposure of procoagulant and antigenic sites on the fiber, which can cause early acute thrombosis, degradation of fibers, and inflammation (Lopera Higuita and Griffiths, 2020). Photooxidation cross-linked the graft’s collagen fibers to cover parts of the exposed antigen sites, which reduced the immune response and the foreign body reaction and prevented rapid cell infiltration and early degradation of the ECM to form an aneurysm or rupture (Lü et al., 2010). However, the degree of the vascular graft’s cross-linking by photooxidation treatment was not sufficient. Our previous studies have also shown that after photooxidation cross-linking of decellularized bovine veins, elastic fiber were degraded, which may reduce its biomechanical properties *in vivo*. The results indicated that moderate cross-linking of photooxidation and PGG can improve the biomechanical properties of vascular scaffolds, but excessive cross-linking can also lead to reduced vascular compliance. Appropriately cross-linked elastin fibers improved the vascular grafts’ compliance, which has been proven to be related to the intimal hyperplasia *in vivo* and can reduce the failure rate of vascular graft (Cocciolone et al., 2018; Thrivikraman et al., 2020). As a commonly used cross-linking agent, PGG cross-links and stabilizes elastin fiber in vascular graft and also possesses anti-inflammatory, antioxidant, and immunomodulatory effects (Fraga et al., 2010; Leopoldini et al., 2011).

In our study, the photooxidation and PGG cross-linking improved the graft’s biomechanical properties, biocompatibility, and stability. The biomechanical results showed that the DOP groups’ compliance, suture retention strength, and resistance to elastase degradation were improved. The residual biomechanical properties, which reflected the stress state in the vascular tissue were important for the vascular wall’s regeneration, reconstruction, and the smooth muscle cell functions’ regulation (Cocciolone et al., 2018). Additionally, the degree of cross-linking and SEM showed that the cross-linking strengthened the fiber connections, reduced the decellularized material’s pore structure, and covered the coagulation and antigen sites caused by the decellularization process. However, excessive cross-linking will cause the vascular graft fibers to be tightly connected and stiff, and the luminal surface’s pore structure will be significantly reduced, which may inhibit cell infiltration and remodeling (Liang et al., 2004). The results of cell adhesion experiments and the cytotoxic MTT test showed that there was no difference between the four groups, indicating that the decellularization process and cross-linking did not increase the vascular graft’s toxicity. The strategy of cross-linking for collagen and elastic fibers improved the performance of decellularized vascular grafts.

The implantation of heterogeneous tissue materials in the body will cause a series of inflammation in the body. Studies have shown that immunogenic heterogeneous tissues can cause a continuous immune rejection in the body and eventually decline (Lopera Higuita and Griffiths, 2020). Hsing Wen Sung’s groups showed that un-cross-linked acellular tissue can cause an inflammatory reaction *in vivo* and can be infiltrated and degraded by inflammatory cells (Liang et al., 2004). Additionally, un-cross-linked and excessive cross-linking of acellular tissues can lead to tissue regeneration failure *in vivo* due to excessive degradation or inhibition of cell infiltration, respectively. However, the process of decellularization will also cause the collagen fibers’ antigen sites to be exposed, causing the infiltration of inflammatory immune cells. An appropriate cross-linking treatment may cover part of the antigenic sites and inhibit the excessive inflammatory response at the early stage *in vivo* while permitting the infiltration of cells to promote the tissue regeneration at the late stage. The HE results showed that the vascular wall was infiltrated by cells in the NA and DC groups, while the cell infiltration in the DOP group was mild, only infiltrating the outer layer’s superficial part and the basement membrane’s surface layer. The IHC results of our rat subcutaneous embedding experiments showed that there were more CD68^+^ macrophage cells and CD3^+^ cells in the NA and DC groups at 4w, indicating that the inflammatory immune response persisted after implantation because the existence of natural BIMA cells and antigen components caused the host’s response. However, the CD68^+^ macrophage cells and CD3^+^ cells were significantly decreased in the DO and DOP groups. This may be due to the photooxidation and PGG cross-linking, which strengthened DC-BIMA’s biomechanical properties and inhibited inflammation *in vivo*. When modifying decellularized materials’ properties, an appropriate cross-linking treatment can effectively improve vascular grafts’ performance *in vivo*. When tissue-engineered blood vessels are implanted in the body, the best reconstruction process is the balance of the degradation rate and regeneration of ECM *in vivo*. Degradation that occurs too quickly can lead to loss of the vascular graft’s biomechanical properties and the formation of aneurysm, especially in the arterial system, which may rupture.

To evaluate the function of the BIMA *in vivo*, the vascular grafts were implanted into the replacement model of the rabbit abdominal aorta. In the DC group, there were two cases of occlusion due to thrombus and two cases of stenosis. There were also two cases of stenosis in the DO group. The results of morphology showed that a large number of cells infiltrated in the adventitia and neointima of the vessel wall, the elastic fibers were fractured and degraded, the structure of the neointima was disorganized, and the regenerated collagen fibers were irregularly arranged in the DC and DO groups. In addition, the exposed basement membrane and fibers might promote the adsorption and activation of fibrinogen, which further activated platelets, resulting in an acute thrombus. However, in the DOP group, there was little cellular infiltration in the adventitia of vascular grafts. The elastic fiber structure was almost intact and elastin preservation was significantly higher in the DC and DO groups. Furthermore, the regenerated collagen fiber structure in the neointima was regular with uniform distribution of cells, which may avoid thrombus and stenosis, resulting to the high patency rate. Photooxidation treatment as well as the PGG cross-linked collagen and elastin fiber and the combination of the two was effective in maintaining matrix integrity, pointing to stabilized arterial scaffolds as viable small-diameter vascular grafts.

*In vivo* calcification of xenogeneic tissue materials was also the cause of vascular transplantation failure. Acellular materials exposed the calcification sites of collagen or elastic fibers *in vivo*, which combined with calcium ions *in vivo*, resulted in calcium deposition (Basalyga et al., 2004; Pai et al., 2011). Additionally, the degradation and fracture of elastic fiber *in vivo* can further expose the calcification sites and aggravate the calcium deposition *in vivo*. Previous studies have shown that PGG cross-linking of biomaterials can prevent early degradation of elastic fibers and inhibit calcification (Pennel et al., 2014; Jiang et al., 2017). In our study, calcium deposition occurred in both the DC and DO groups at 4 weeks. EVG results also showed that the elastic fibers of BIMA in the DOP group were almost similar to those before implantation, while the elastic fibers in the DO and DC groups were broken and degraded. The results showed that PGG treatment prevented the early degradation of elastic fibers and may be contributed to inhibit calcification *in vivo*. Thus, to hinder calcification, it was necessary to treat acellular vessels with decalcification and prevent the fiber degradation.

In the current research, the inflammatory response of biomaterial was closely related to tissue regeneration *in vivo*. An excessive inflammatory response can lead to a foreign body reaction and degradation of biomaterials (Umashankar et al., 2013; Aamodt and Grainger, 2016). The study demonstrated that photooxidation and PGG cross-linking inhibited the infiltration of CD68^+^ macrophage cells in the graft, promoted the regeneration of host SMC, and remodeled neointima. Similar to the results of subcutaneous implantation in rats, in the DC and DO groups, the CD68^+^ macrophages were present in the neointima and grafts, suggesting that partial degradation occurs in the early stage of vascular grafts, exposing part of the antigenic sites and promoting inflammatory cell infiltration, which may lead to vascular calcification, expansion, or rupture in the later stage of implantation. The appearance of calponin^+^ and α-SMA^+^ cells in the neointima, accompanied by the infiltration of vimentin^+^ cells, suggested that host cells infiltrated and differentiated into vascular smooth muscle cells and fibroblasts within the vascular scaffold, leading to early remodeling of the grafts. In the DOP group, there were no CD68^+^ cells in the neointima, less cell infiltration in the vascular wall and less elastin degradation, indicating that the photooxidation and PGG cross-linking might prevent the early degradation of vascular scaffolds and inhibit the inflammatory response. The CD206^+^ M2 macrophages cells and calponin^+^/α- SMA^+^ double positive cells were found in the neointima, suggesting that functional type smooth muscle cells appeared in the grafts might initiate the vascular invasion early phase of remodeling and regeneration. It may be that PGG has an anti-inflammatory effect, which enhanced the effect of anti-inflammatory cells and accelerated the resolution of inflammation. Previous studies have shown that an increase in type I collagen fibers can cause intimal hyperplasia with a corresponding decrease in the content of type I collagen fibers (Purushothaman et al., 2011). In our study, the ratio of relative content of type I/III collagen fibers in the DOP group was significantly higher than that in the DO and DC groups, which indicated that the regenerated collagen fibers in the neointima were accelerated into type I fibers and improved the patency. Although there was a monolayer of cell coverage on the luminal surface of blood vessels in the groups, there was no CD31 positive cell adhesion, suggesting that vascular grafts had no regeneration of endothelial cell at 4w.

Previous studies have shown that xenogeneic biomaterials can form neointima after implantation *in vivo*, but its composition are still unknown (Pennel et al., 2013; Pennel et al., 2014). Our study was the first to analyze the neointima by mass spectrometry to further understand the composition and mechanism of decellularized small-diameter vascular xenograft remodeling in a rabbit abdominal aortic replacement model at 4w. The mass spectrometry analysis showed that neointima contained similar protein species in the three groups. The protein components are mainly plasma-related proteins, cytoskeleton proteins, glycoproteins, and secreted factors. Due to the species of experimental animals, we only identified a small number of proteins and failed to find statistically significant difference proteins. In the groups, the composition of the neointima was mainly fibrinogen, hemoglobin, albumin, vitronectin, and IgG from blood components, maybe due to the fact that the decellularization treatment exposed the basement membrane and a significant number of collagen fibers, leading to multiple protein adsorption in plasma, cell recruitment, and platelet activation, which caused excessive inflammatory response, thrombosis, and failure to resolve in time at the early stage. Although no statistically significant differential proteins were found among the three groups, the results of IHC, Sirius Red, and Masson stains showed that the neointima quickly achieved a stable structure in the DOP group, which indicated that modification of the grafts may alter the remodeling of the neointima. Interestingly, we preliminarily analyzed that in the neointima, plasma proteins constitute the major component, which may be related to the process of protein adsorption, with large amounts of albumin, platelets, fibrinogen, hemoglobin, and immunoglobulins adsorbed on the luminal surface of small-diameter vascular grafts at the early stage (Othman et al., 2018). Protein adsorption on the biomaterial surface is one of the very first events in the interaction between materials and blood, which is a determinant for the subsequent processes of cell growth, differentiation, and extracellular matrix formation (Yang et al., 20142014; Swartzlander et al., 2015). Further observations and studies are essential to the relationship between protein adsorption and the neointimal regeneration and remodeling of decellularized vascular grafts. This will help in understanding the regeneration mechanism of small-diameter vascular xenografts *in vivo* and provide new strategies for designing better tissue engineering vascular grafts.

Finally, we recommend that a more effective decellularization process should be used to completely remove cellular and most of antigen components to preserve a good three-dimensional structure and to obtain a potentially homogenous or heterogeneous decellularized small-caliber vascular graft for clinical use. Due to the decellularization process tend to alter the structure of ECM and expose the basement membrane, fibers and antigen sites, the decellularized grafts require an appropriate cross-linking, which can improve biomechanical properties and biocompatibility.

### Limitations of the Study

The study’s main limitation was that the implantation time of the rabbit abdominal aorta model was too short, and the long-term patency rate and the remodeling situation still need further research. Additionally, no further modifications were made in the graft to promote the regeneration of endothelial cells. Before clinical application, small-caliber acellular material vascular grafts still need to be studied and observed in a large animal model.

## Conclusion

The procedure of decellularization by sonication treatment and perfusion can effectively remove the cellular components and α-gal antigen of BIMA and can preserve a good three-dimensional structure. The decellularized BIMA cross-linked by photooxidation and PGG improved the biomechanical properties and biocompatibility of the grafts, including the reduction of inflammatory response, the protection of elastic fibers, the remodeling of the neointima, and the inhibition of calcification, thereby resulted in high patency. Modified BIMA is a promising candidate for constructing tissue engineering vascular graft.

## Data Availability

The raw data supporting the conclusion of this article will be made available by the authors, without undue reservation.
