# Supplementary material for: Photooxidation and Pentagalloyl Glucose Cross-Linking Improves the Performance of Decellularized Small-Diameter Vascular Xenograft In Vivo
Source: Front Bioeng Biotechnol. 2022 Mar 24;10:816513. doi: 10.3389/fbioe.2022.816513 (PMC8987116; doi:10.3389/fbioe.2022.816513)

**Supplementary Materials for**

**Photooxidation and penta-galloyl glucose crosslinking improves the remodeling of neointimal of decellularized small diameter vascular xenograft in vivo**

Histology:

The kit was purchased in Servicebio.

Immunohistochemistry

For immunohistochemical analysis, sections were dewaxed and rehydrated using xylene followed by a decreasing series of ethanol. Endogenous peroxidases were quenched by incubating the slides in 0.6% methanol-hydrogen peroxide solution for 15 minutes at room temperature. After the slides were left to cool down to room temperature, unspecific binding sites were blocked by incubating the slides in 1.5% normal goat serum for 30 minutes at room temperature. Primary antibodies were diluted in PBS and incubated over night at 4℃, followed by washing steps in PBS and incubation with the corresponding secondary antibodies. DAB/AEC-Solution was used to detect positive signals. Sections were counterstained using Hematoxylin and dehydrated using a graded series of ethanol and xylene(AEC-solution can not dehydrate using alcohol).

Immunofluorescence

Briefly, sections were dewaxed in xylene and rehydrated using a decreasing series of ethanol. Endogenous peroxidase activity was blocked by incubating the slides in 3% methanol. Antigen retrieval was performed by incubating the sections in 0,01M citrate buffer for 30 minutes in a steamer. After cooling the sections to room temperature, a protein block (10% normal goat serum) was used to minimize unspecific binding of the primary antibody. Then follow the steps below.

1. Add primary antibody: gently shake off the blocking solution, drop the primary antibody prepared by PBS in a certain proportion on the slices, mix the two primary antibodies in a certain dilution proportion, and drop them on the tissues. The slices are placed flat in the wet box and incubated at 4℃ overnight.
2. Add secondary antibody: put the glass slide in PBS (pH7.4), shake and wash it on the decolorization shaking table for 3 times, each time for 5min. After the slices were slightly dried, the secondary antibody covered tissues matched with the corresponding species and genus markers of the primary antibody were added in the circle, and incubated at room temperature for 50 min. (two kinds of secondary antibodies are mixed and incubated in a certain proportion)

3. DAPI counterstaining nuclei: after the sections were slightly dried, DAPI staining solution was added in the circle and incubated at room temperature for 10 minutes.

4. Autofluorescence quenching: after the slices are slightly dried, add autofluorescence quenching agent into the circle for 5min and rinse with running water for 10min.

5. Sealing slide: place the slide in PBS (pH7.4), shake and wash it on the decolorization shaking table for 3 times, each time for 5min. After the slices were slightly dried, they were sealed with anti fluorescence quenching sealing agent.

6. Microscopic examination and photographing: the slices are placed under the scanner to collect images.

Supplemental Figures:

Fig.1.Comparison of the Compliance (A), Elastinase (B) and Fixation index (C) test of three different concentrations of PGG(0.1%, 0.25%, 0.5%) in DOP group. The resistance to elastinase and fixation index of 0.5% DOP group was significantly higher than that other two groups. In the test of diametrical compliance,with the increase in concentration of PGG, the compliance were first significantly increased and then decreased. (*p<0.05, ***p<0.001, Kruskal–Wallis test with Tukey's post hoc test)

Fig.2. Seven days after seeding of umbilical vein endothelial cells(EAhy926) on vascular sheets, HE and immunohistochemical staining for CD31, an endothelial cell marker, were performed, and endothelial cells continuously covered the luminal surface of the vascular sheets.

Fig.3. Immunohistochemical results of the four groups of subcutaneous transplantation specimens. The results showed the presence of α-SMA^+^ cells in the grafts.

Fig.4. Elastic Van-Gieson (EVG) staining for elastin and quantification of relative elastin content from the histological images (A) in the DC, DO, and DOP groups after implantation into rabbits for 4 w. Pre-DC: EVG staining of decellularized BIMA before implantation in vivo (***p<0.001, Kruskal–Wallis test with Tukey's post hoc test).

Fig.5. Quantification of the number of CD68^+^ cells in the DO and DOP groups from the rabbit model; the number of positive cells in the DC group was significantly more than that of the DO group. CD68^+^ cells were not detected in DOP group (***p<0.001, Mann - Whitney U test).

Fig.1


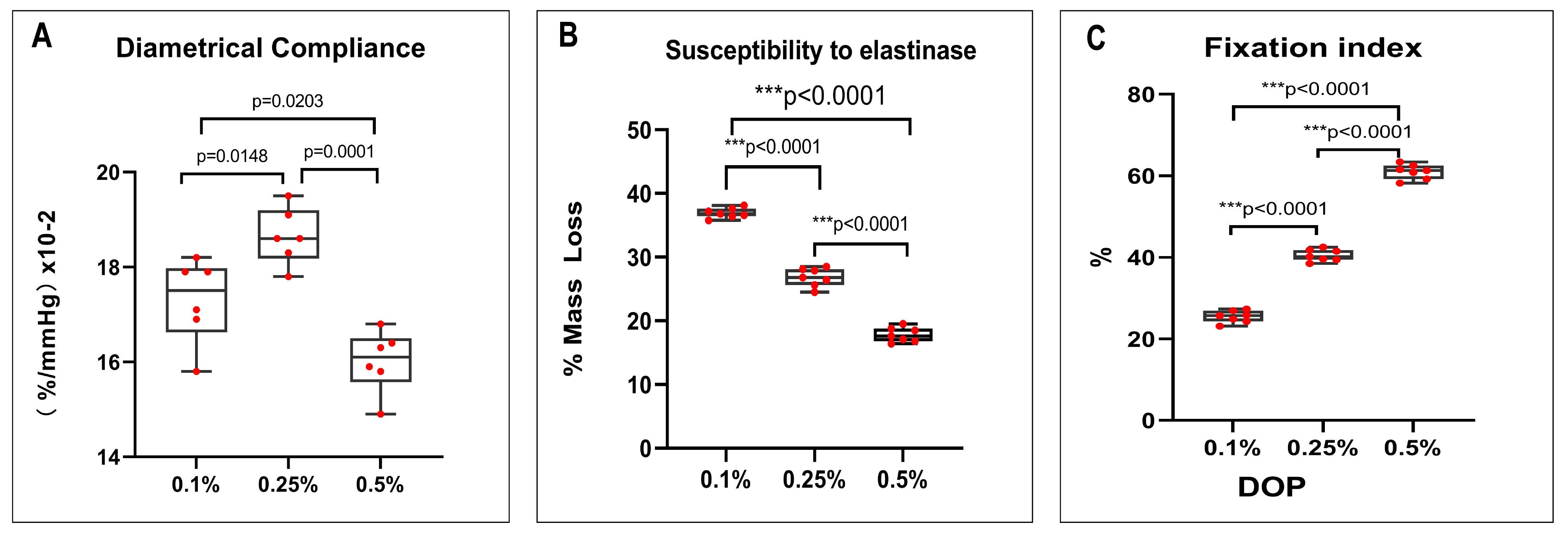


Fig.2


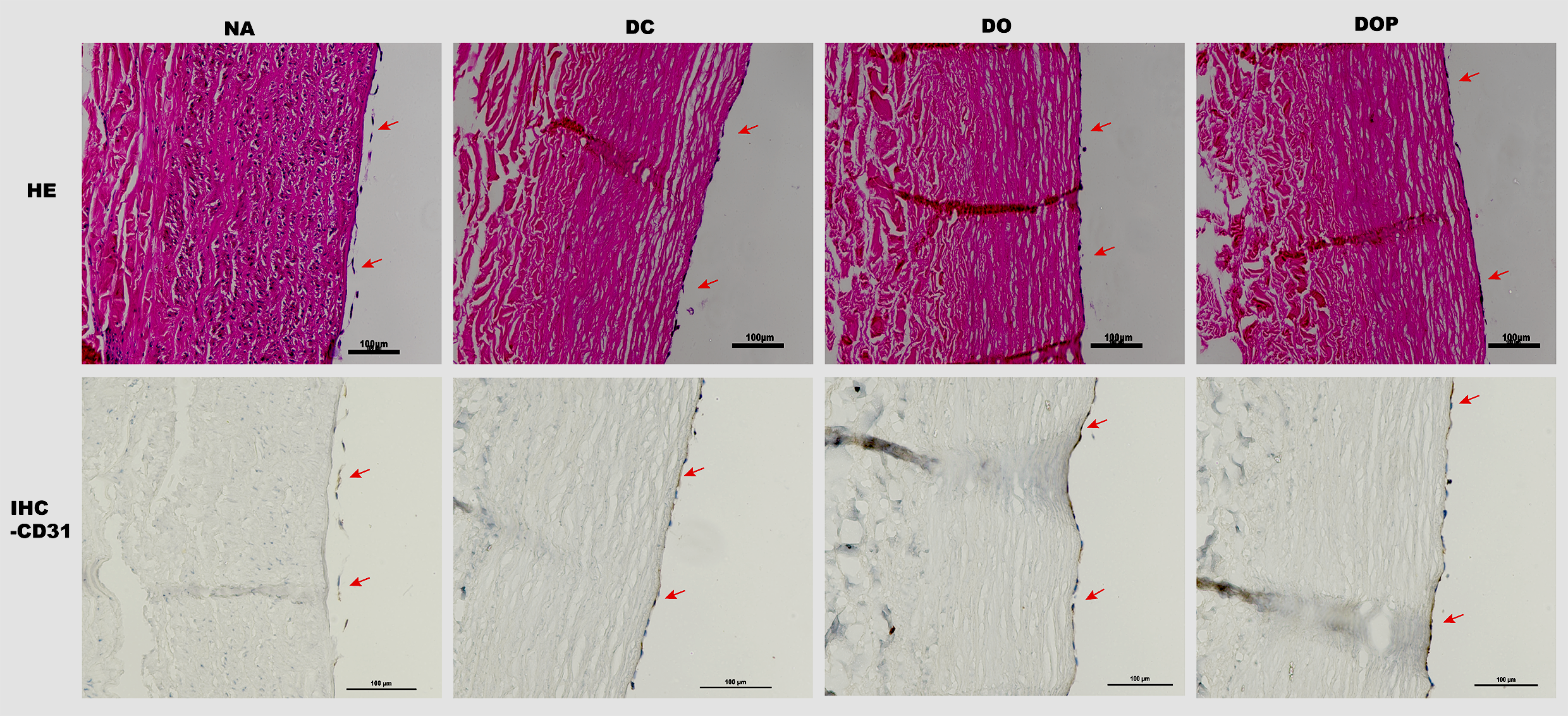


Fig.3


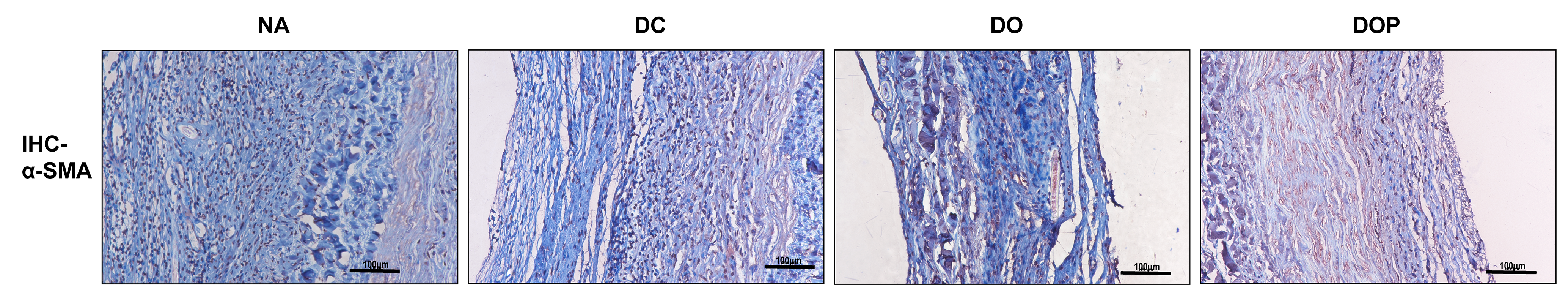


Fig.4


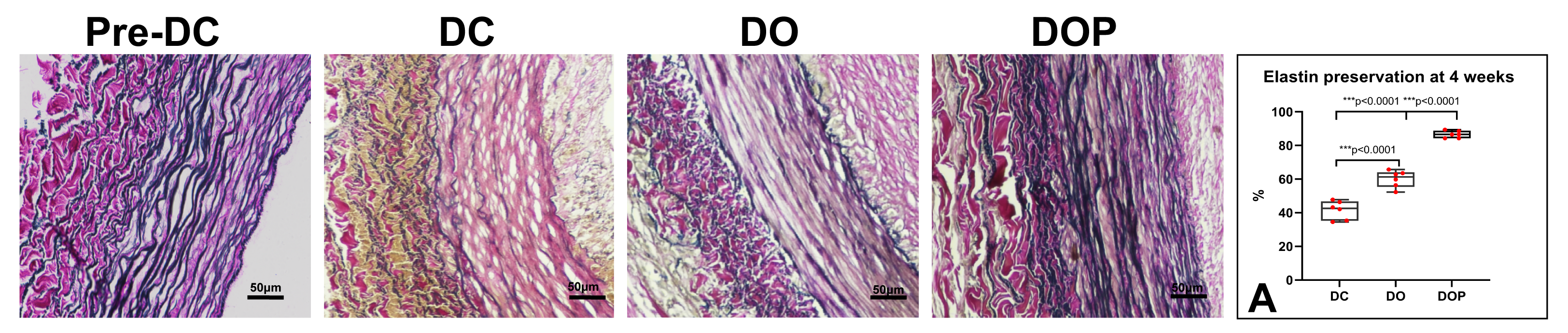


Fig.5


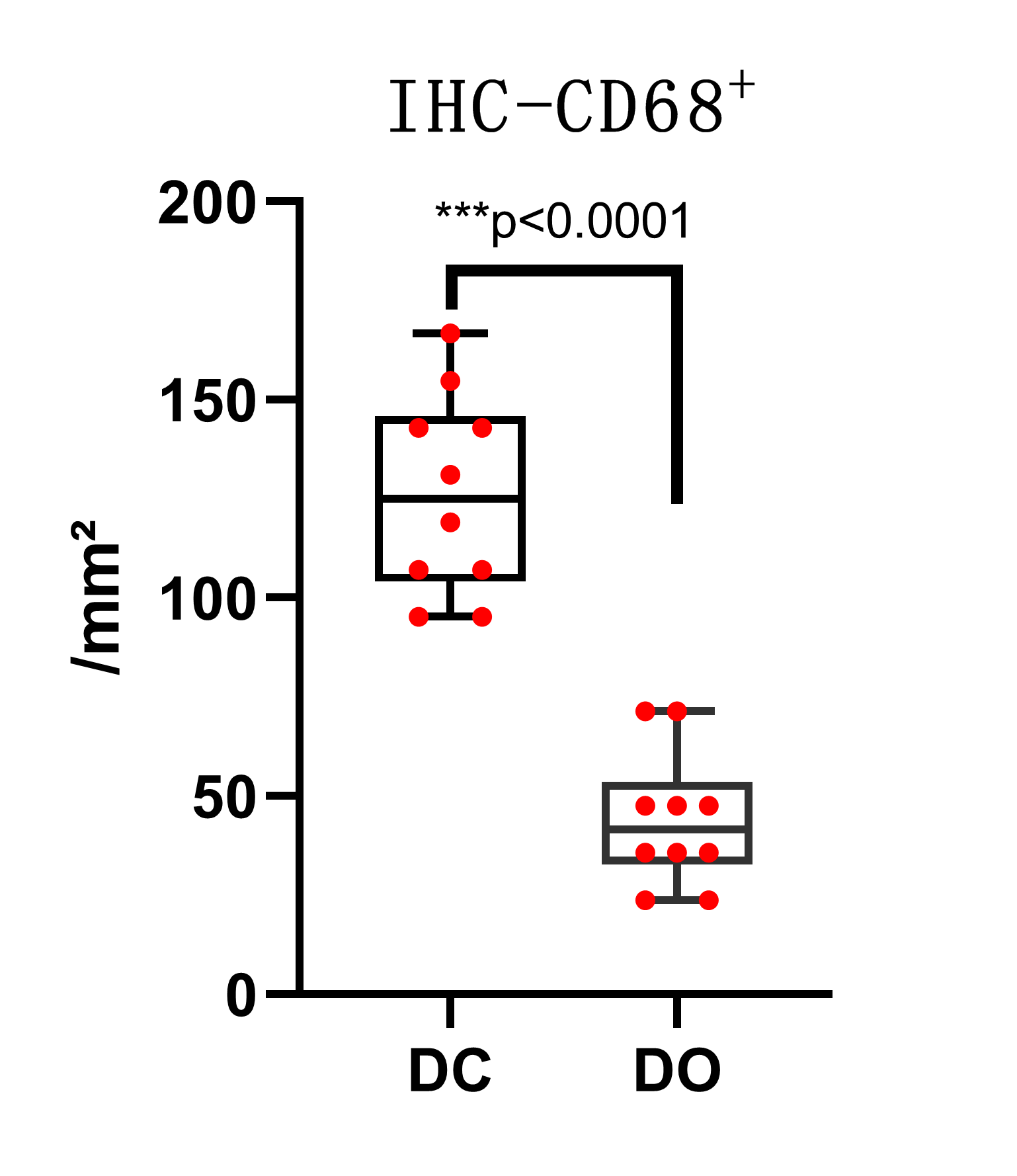

Supplement: Supplementary file 1 [file DataSheet1.doc]
