# Supplementary material for: Photooxidation and Pentagalloyl Glucose Cross-Linking Improves the Performance of Decellularized Small-Diameter Vascular Xenograft In Vivo
Source: Front Bioeng Biotechnol. 2022 Mar 24;10:816513. doi: 10.3389/fbioe.2022.816513 (PMC8987116; doi:10.3389/fbioe.2022.816513)
Supplement: Supplementary file 2 [file DataSheet2.doc]

**Supplementary material for mass spectrometry data**

Table 1. Top 30 proteins in differential protein analysis of the two groups with the DO and DC groups. (The limma package in the R language was used for difference analysis)

| Sample | logFC | t | P.Value | adj.P.Val |
| --- | --- | --- | --- | --- |
| HPX | 0.974907842 | 4.536755188 | 0.003411519 | 0.631130938 |
| GC | -0.430609516 | -3.222471821 | 0.016670185 | 1 |
| TF | 0.459282551 | 3.073771426 | 0.020274911 | 1 |
| CCT6 | 0.174042374 | 2.645476933 | 0.036219049 | 1 |
| VDAC1 | -0.071877292 | -2.389051046 | 0.051763914 | 1 |
| HBB1 | -0.388796013 | -2.385340769 | 0.05203424 | 1 |
| EIF4A1 | -0.310026062 | -2.342278719 | 0.055280917 | 1 |
| TPI1 | 0.566599258 | 2.280917079 | 0.060273249 | 1 |
| APOA1 | 0.555706226 | 2.156155739 | 0.071902293 | 1 |
| BPI | -0.524580845 | -2.02989429 | 0.08600436 | 1 |
| ALDH1A1 | -0.415614253 | -1.932485719 | 0.098758335 | 1 |
| TPM2 | 0.333359893 | 1.90876575 | 0.102139083 | 1 |
| PON1 | 0.485418175 | 1.87872393 | 0.106585666 | 1 |
| VDAC2 | -0.114113981 | -1.863741199 | 0.108874145 | 1 |
| S100A12 | -0.359377883 | -1.836432056 | 0.11317034 | 1 |
| OGN | 0.809523118 | 1.800237359 | 0.11912121 | 1 |
| EEF1G | 0.170975245 | 1.794141589 | 0.120153058 | 1 |
| ANXA8 | 0.106056738 | 1.782166227 | 0.122205575 | 1 |
| AHSG | -0.365389785 | -1.712454489 | 0.134846257 | 1 |
| FKBP4 | 0.199718608 | 1.66588161 | 0.143983305 | 1 |
| HSP90B1 | 0.433287454 | 1.657674719 | 0.145653605 | 1 |
| ACTA1 | 0.248203759 | 1.643224536 | 0.148639544 | 1 |
| HSP90AA1 | -0.219618053 | -1.617083189 | 0.154189698 | 1 |
| ALB | 0.326002001 | 1.542098532 | 0.171215051 | 1 |
| HP | 0.686230367 | 1.492915162 | 0.183319482 | 1 |
| ANXA1 | -0.393729263 | -1.458328315 | 0.192300083 | 1 |
| EIF5A | 0.162276601 | 1.412249721 | 0.204890541 | 1 |
| C3 | 0.354301491 | 1.397284584 | 0.209137935 | 1 |
| ENO3 | 0.324338754 | 1.293288831 | 0.240901181 | 1 |
| MMP1 | 0.176037438 | 1.225740205 | 0.263742316 | 1 |

Table 2. Top 30 proteins in differential protein analysis of the two groups with the DOP and DO groups. (The limma package in the R language was used for difference analysis)

| SAMPLE | logFC | t | P.Value | adj.P.Val |
| --- | --- | --- | --- | --- |
| GC | 0.500675717 | 3.746813131 | 0.008578971 | 0.873501572 |
| TPM1 | -0.307795383 | -2.449871067 | 0.047534841 | 0.873501572 |
| CRP | -0.862189596 | -2.376224858 | 0.052704652 | 0.873501572 |
| PKM | 0.291837246 | 2.260139577 | 0.062067083 | 0.873501572 |
| WARS1 | -0.159290902 | -2.253817806 | 0.062623691 | 0.873501572 |
| CKM | 0.63593403 | 2.184138241 | 0.069108576 | 0.873501572 |
| FMOD | -0.26259004 | -2.169631955 | 0.070542771 | 0.873501572 |
| ADH5 | 0.872277744 | 2.158765189 | 0.071636977 | 0.873501572 |
| ANXA1 | 0.578404608 | 2.142344744 | 0.073323261 | 0.873501572 |
| SAA2 | -0.232923158 | -2.005745807 | 0.089003805 | 0.873501572 |
| TGFBI | 0.294496641 | 1.993629702 | 0.090547989 | 0.873501572 |
| CAPZA2 | 0.303438788 | 1.936926307 | 0.098137878 | 0.873501572 |
| EEF1G | 0.176499556 | 1.852111364 | 0.11068377 | 0.873501572 |
| FLNB | 0.151791643 | 1.808838946 | 0.117679885 | 0.873501572 |
| CYB5A | 0.305632203 | 1.793442492 | 0.120271951 | 0.873501572 |
| CAST | 0.410862996 | 1.783178842 | 0.122030706 | 0.873501572 |
| CORO1B | 0.200468995 | 1.783139967 | 0.122037415 | 0.873501572 |
| CYCS | 0.195930998 | 1.783137577 | 0.122037827 | 0.873501572 |
| C8G | -0.130163668 | -1.783069973 | 0.122049495 | 0.873501572 |
| B2M | 0.127546342 | 1.783064956 | 0.122050361 | 0.873501572 |
| PPP2CA | -0.110619589 | -1.78302344 | 0.122057526 | 0.873501572 |
| CD14 | 0.108785442 | 1.783017743 | 0.12205851 | 0.873501572 |
| PPP2CB | -0.070810598 | -1.78278218 | 0.122099177 | 0.873501572 |
| AKR1C5 | -0.052831545 | -1.782456793 | 0.122155372 | 0.873501572 |
| PPP1CB | 0.052419753 | 1.78244522 | 0.122157372 | 0.873501572 |
| TPT1 | 0.021952833 | 1.778951294 | 0.122762383 | 0.873501572 |
| HP | -0.787814751 | -1.713915098 | 0.134568903 | 0.908971822 |
| TXN | -0.308376776 | -1.677105296 | 0.141728603 | 0.908971822 |
| YWHAQ | 0.795304404 | 1.649695257 | 0.14729532 | 0.908971822 |
| ITIH3 | 0.17332125 | 1.638477822 | 0.149633034 | 0.908971822 |

Table 3. Top 30 proteins in differential protein analysis of the two groups with the DOP and DC groups. (The limma package in the R language was used for difference analysis)

| sample | logFC | t | P.Value | adj.P.Val |
| --- | --- | --- | --- | --- |
| TF | 0.63566812 | 4.254240666 | 0.004693601 | 0.614111137 |
| EEF1G | 0.347474801 | 3.646252952 | 0.009713319 | 0.614111137 |
| APOA1 | 0.921986525 | 3.57733357 | 0.010585646 | 0.614111137 |
| HPX | 0.730289581 | 3.398418703 | 0.013278079 | 0.614111137 |
| VDAC1 | -0.095925981 | -3.18837922 | 0.017430594 | 0.644931989 |
| CCT6 | 0.161606326 | 2.456446652 | 0.047099678 | 1 |
| TPI1 | 0.586550905 | 2.361234961 | 0.053826593 | 1 |
| CAP18 | -0.274090204 | -2.309576548 | 0.057886073 | 1 |
| EZR | 0.599210853 | 1.917839025 | 0.100832554 | 1 |
| ITIH3 | 0.202211411 | 1.911588524 | 0.101730824 | 1 |
| HBB1 | -0.298741528 | -1.832838619 | 0.113747892 | 1 |
| TGFBI | 0.267245116 | 1.809147297 | 0.117628533 | 1 |
| S100A9 | -0.33591773 | -1.783172817 | 0.122031745 | 1 |
| FTH1 | -0.18965443 | -1.783133983 | 0.122038447 | 1 |
| APOD | 0.089332383 | 1.782934094 | 0.122072949 | 1 |
| PPP1CA | 0.062313023 | 1.782663132 | 0.122119734 | 1 |
| CYB5A | 0.291635064 | 1.711307609 | 0.135064423 | 1 |
| LDHA | 0.520541498 | 1.682294232 | 0.14069766 | 1 |
| PON1 | 0.431257931 | 1.669106426 | 0.143331994 | 1 |
| FGB | -0.874817821 | -1.664214102 | 0.144321197 | 1 |
| HSP90AA1 | -0.222560713 | -1.63875047 | 0.149575798 | 1 |
| BPI | -0.416314478 | -1.610951656 | 0.155519645 | 1 |
| YWHAQ | 0.775648133 | 1.608922371 | 0.155962187 | 1 |
| VDAC2 | -0.092248487 | -1.506627886 | 0.179867236 | 1 |
| S100A12 | -0.280030736 | -1.43096569 | 0.199688732 | 1 |
| CRP | -0.512435959 | -1.41229153 | 0.204878785 | 1 |
| CAPN2 | 0.549166671 | 1.395890333 | 0.209537677 | 1 |
| PKM | 0.177940037 | 1.378060286 | 0.214710771 | 1 |
| ALDH1A1 | -0.296036494 | -1.376483823 | 0.215173641 | 1 |
| P4HB | 0.195778901 | 1.375117451 | 0.21557555 | 1 |
